# Acute gastrointestinal injury and altered gut microbiota are related to sepsis-induced cholestasis in patients with intra-abdominal infection: a retrospective and prospective observational study

**DOI:** 10.3389/fmed.2023.1144786

**Published:** 2023-07-27

**Authors:** Beiyuan Zhang, Xiancheng Chen, Chenhang He, Ting Su, Ke Cao, Xiaoyao Li, Jianfeng Duan, Ming Chen, Zhanghua Zhu, Wenkui Yu

**Affiliations:** ^1^Department of Critical Care Medicine, Affiliated Drum Tower Hospital, Medical School of Nanjing University, Nanjing, Jiangsu Province, China; ^2^Nanjing Drum Tower Clinical College of Xu Zhou Medical University, Nanjing, China

**Keywords:** intra-abdominal infection, sepsis-induced cholestasis, acute gastrointestinal injury, gut microbiota, 16S ribosomal ribonucleic acid sequencing

## Abstract

**Background:**

Sepsis-associated liver dysfunction (SALD) has high incidence and mortality in patients with intra-abdominal infection (IAI). The associations between acute gastrointestinal injury (AGI), gut microbiota, and SALD were evaluated in patients with IAI.

**Methods:**

A retrospective study was conducted to assess the relationship between AGI and SALD in patients with IAI. Patients were divided into non-SALD and sepsis-induced cholestasis (*SIC*) groups, which is a subtype of SALD. *SIC* was defined as total bilirubin >2 mg/dL. AGI incidences between the two groups were compared using Chi-square test. Subsequently, a prospective study was conducted to investigate the gut microbiota differences between patients without SALD and those with *SIC*. Fecal samples were collected on days 1, 3, and 7 after admission to analyze changes in gut microbiota using 16S ribosomal ribonucleic acid sequencing.

**Results:**

One hundred thirty-four patients with IAI were included retrospectively, with 77 SALD and 57 non-SALD cases. Among patients with SALD, 71 were diagnosed with *SIC*. Patients with *SIC* had a higher incidence of AGI compared to those without SALD (28.07% vs. 56.34%, *p* < 0.05), and a severity-dependent relationship was found between AGI grade and *SIC* occurrence. Subsequently, 20 patients with IAI were recruited prospectively, with 10 patients each assigned to the non-SALD and *SIC* groups. Patients with *SIC* had a more severe gut microbiota disorder on day 7 than those without SALD, including lower microbiota diversities, decreased abundance of *Firmicutes* and *Bacteroidetes,* and increased abundance of *Proteobacteria* and *Actinobacteria* at the phylum level. Furthermore, *Burkholderia − Caballeronia − Paraburkholderia* and *Delftia,* the two most abundant genera, were significantly higher in the *SIC* group than in the non-SALD group. Functional prediction analysis showed that the top three KEGG pathways were ribosome, pyrimidine metabolism, and the two-component system. During the first week, the abundance of *Proteobacteria* decreased significantly, whereas *Cyanobacteria* increased in the non-SALD group; however, the phyla taxa did not change significantly in the *SIC* group.

**Conclusion:**

There exists a severity-dependent relationship between AGI grade and *SIC* occurrence in adult patients with IAI. A severe gut microbiota disorder was discovered in *SIC* during the first week of the intensive care unit stay.

## 1. Introduction

The Sepsis 3.0 consensus defines sepsis as a life-threatening organ dysfunction caused by the dysregulated host response to infection (1). The liver, an important immune and metabolic organ, plays a crucial role in host defense against invading pathogens and endotoxins and in maintaining metabolic and immunological homeostasis. Studies indicate that sepsis-associated liver dysfunction (SALD) substantially impacts the severity and prognosis of sepsis (2, 3).

Intra-abdominal infection (IAI) is the second leading source of infection for sepsis after pneumonia in intensive care units (ICU) (4) and is often related to high morbidity and mortality rates (5, 6). The incidence of SALD in patients with IAI is considerably higher than that of the general population with sepsis (3, 7). However, the pathogenesis of SALD remains unclear. Nevertheless, the unique anatomical structure of the liver enables a close association with the gut, and increasing evidence indicates that the gut microbiota and related metabolites are associated with various liver diseases (8). It is interesting to note that the incidence of acute gastrointestinal injury (AGI) in patients with IAI was also considerably higher than in patients with other site infections, and the AGI degree was more serious according to guidelines proposed by the European Society of Intensive Care Medicine (ESICM) in 2012 (9, 10). An animal experiment revealed that mice with SALD exhibit more severe gut microbiota dysbiosis than the control group (11). In addition, mice that received fecal microbiota transplantation from sepsis-sensitive mice, defined as those with a survival time of approximately 24 h after cecal ligation and puncture, exhibited more severe liver dysfunction compared to sepsis-resistant mice, defined as those with a survival time of 7 days (12). In contrast, modulating gut microbiota through pharmacological interventions or fecal microbiota transplantation has been demonstrated to alleviate liver dysfunction in septic mice (11, 13). However, relevant clinical trials on this topic are scarce.

The study aimed to explore the relationships between AGI, gut microbiota, and SALD in patients with IAI, which could provide a potentially efficacious treatment option. A single-center retrospective observational study was performed to investigate the relationship between AGI and sepsis-induced cholestasis (*SIC*), a subtype of SALD, among patients with IAI. Subsequently, a prospective study was conducted to analyze and compare the diversity and composition of gut microbiota in patients with IAI without SALD or with *SIC*, respectively. The dynamic changes in the gut microbiota were also investigated within 1 week after ICU admission.

## 2. Patients and methods

### 2.1. Study participants

The study protocol was approved by the Medical Ethics Committee of Nanjing Drum Tower Hospital (number: 2020-012-01). Informed consent was obtained from all patients or immediate family members. The research was carried out in accordance with the Declaration of Helsinki.

The retrospective observational study was conducted at the Nanjing Drum Tower Hospital between January 2019 to March 2022. Adult patients (≥18 years) with IAI who met the criteria for Sepsis 3.0 were identified. The following exclusion criteria applied:

1. discharge or death within 72 h after ICU admission;
2. serious chronic liver diseases, such as decompensated cirrhosis and end-stage liver cancer;
3. hospitalization due to primary hepatobiliary diseases, such as trauma, hepatitis, or cholelithiasis;
4. other causes of liver injury, such as drugs and poisons; and
5. pregnancy.

The enrolled patients were assigned to the non-SALD and SALD groups based on whether SALD was present during their ICU stay. Then, patients with SALD were further classified into hypoxic hepatitis and *SIC*.

### 2.2. Data collection and outcomes

The following data were collected within 24 h after ICU admission:

1. demographic information: age, sex, and comorbidity;
2. disease severity scores: Acute Physiology and Chronic Health Evaluation (APACHE) II score; Sequential Organ Failure Assessment (SOFA) score;
3. the primary site of abdominal infection: upper or lower gastrointestinal tract, pancreas, and other sites; and
4. Laboratory indicators: white blood cell (WBC) count, neutrophil count, lymphocyte count, monocyte count, platelet, C-reactive protein (CRP), procalcitonin, human leukocyte antigen-DR (HLA-DR), and albumin.

The following information during the ICU stay was also collected:

1. complications: respiratory failure, septic shock, acute kidney injury (AKI), and AGI;
2. absence or presence of bacteremia, pathogens of ascites; and
3. treatment received: mechanical ventilation, vasopressor therapy, renal replacement therapy, parenteral nutrition (PN), and antibiotics.

Prognostic indicators, including ICU length of stay (LOS), ICU mortality, and 60-day mortality, were also recorded.

### 2.3. Definitions

SALD was diagnosed when any of the following criteria were met during the ICU stay (14):

1. Serum aminotransaminase levels >20 times the upper limit of normal (> 800 IU/L), including alanine aminotransferase (ALT) and aspartate aminotransferase (AST); and
2. Total bilirubin (TBIL) > 2 mg/dL.

Hypoxic hepatitis is defined as elevated transaminase (> 800 IU/L) only (14). *SIC* is diagnosed when TBIL >2 mg/dL appears (14). IAI was diagnosed according to the 2005 International Sepsis Forum Consensus Conference (15). The patient presented with abdominal symptoms and signs, such as fever (≥ 38°C), tenderness, and rebound pain, with elevated peripheral blood inflammatory indicators and positive culture of intra-abdominal specimens. Sepsis and septic shock were diagnosed based on the 2016 updated sepsis and septic shock guidelines (1). Sepsis was diagnosed when there was a definite or highly suspected infectious focus and SOFA score ≥ 2 at 24 h after ICU admission. Septic shock was defined as the presence of persistent hypotension, such as systolic blood pressure < 90 mmHg, mean arterial pressure < 65 mmHg, or blood lactate level ≥ 2 mmol/L, even after adequate fluid resuscitation and a need for vasopressors to maintain mean arterial pressure ≥ 65 mmHg in patients with sepsis. AKI was diagnosed according to the Kidney Disease Improving Global Outcomes clinical practice guidelines (16). AGI diagnosis and grading were based on the 2012 ESICM recommendations (10). Grade I: there was a risk of gastrointestinal dysfunction or failure, characterized by transient or self-limiting gastrointestinal symptoms or signs such as nausea, vomiting, mild bloating, diarrhea, gastric retention (≥ 200 mL/day), and absent or diminished bowel sounds (< 3 times/min). Grade II: gastrointestinal dysfunction, characterized by severe gastric retention (≥ 250 mL/day) or reflux, intra-abdominal hypertension (bladder pressure of 12–15 mmHg), gastrointestinal bleeding, etc. Target caloric intake cannot be achieved solely through enteral nutrition; PN support is necessary. Gastrointestinal dysfunction requires medical intervention but does not lead to other organ dysfunctions. Grade III: gastrointestinal failure, characterized by persistent deterioration of gastrointestinal function despite the aforementioned treatments, including elevated intra-abdominal pressure (bladder pressure of 16–20 mmHg) or the development of multiple organ dysfunction syndrome. Grade IV: gastrointestinal failure with concomitant distant organ dysfunction, characterized by the progressive deterioration of gastrointestinal function leading to the worsening of multiple organ dysfunction syndrome and shock. This life-threatening condition requires immediate surgical intervention to reduce intra-abdominal pressure.

### 2.4. Gut microbiota study

A prospective study was conducted in our center between March and September 2022 to further clarify the relationship between gut microbiota and *SIC*. Each participant or relative signed an informed consent form before enrolling in the study. Eligible patients were required to fulfill the same criteria set out above. Additional exclusion criteria were:

1. participation in another clinical trial;
2. previous enterostomy surgery; and
3. chronic inflammatory bowel disease.

All eligible patients were treated with antibiotic and other supportive therapies to maintain organ function. Control of the source of infection was achieved by puncture drainage or surgery, as necessary. Fecal samples were collected for each patient on days 1, 3, and 7 after ICU admission. Other data were collected as described above, and related diseases were defined as previously described. The following exclusion criteria also applied: (1) discharge or death within the first week of ICU admission; (2) a decision by the patient or the patient’s relatives to withdraw from the study; and (3) deviation from the protocol for any reason.

### 2.5. 16S ribosomal ribonucleic acid (rRNA) sequencing

Fecal samples were collected using sterile tubes and then stored in the refrigerator at −80°C. Total genomic deoxyribonucleic acid (DNA) was extracted from these samples using the E.Z.N.A.^®^ Stool DNA Kit (D4015, Omega, Inc., United States) according to the manufacturer’s instructions, eluted in 50 μL of elution buffer and stored at −80°C for subsequent analysis. The hypervariable V3–V4 region of the bacterial 16S rRNA gene was amplified using PCR, forward primer 341F (5′-CCTACGGGNGGCWGCAG-3′), and reverse primer 805R (5′-GACTACHVGGGTATCTAATCC-3′). The PCR products were initially purified using AMPure XT beads (Beckman Coulter Genomics, Danvers, MA, United States) and subsequently quantified using a Qubit (Invitrogen, United States). The amplicon library was evaluated and quantified using an Agilent 2100 Bioanalyzer (Agilent, United States) and Illumina Library quantitative Kits (Kapa Biosciences, Woburn, MA, United States), respectively. Illumina NovaSeq PE250 platform was used for paired-end sequencing of the amplicon library by LC-Biotechnology Co., Ltd. (Hangzhou, China). After sequencing, the raw data were obtained and spliced by overlap. Subsequently, high-quality clean data through quality control and chimera filtering were obtained. After dereplication using the divisive amplicon denoising algorithm, amplicon sequence variants were used to construct the operational taxonomic units (OTUs) table using QIIME 2 to obtain the feature table and sequences for further analysis, including diversity, species taxonomic annotation, and difference analysis. The number of OTUs corresponding to each sample equals the features in the feature sequence.

Alpha and beta diversities were assessed by randomly normalizing them to the same sequences. Next, the feature abundance was normalized using the relative abundance of each sample according to the SILVA (release 132) classifier. The alpha diversity of samples was used to assess species diversity using five indices: observed OTUs, Chao1, Shannon, Simpson, and Pielou_e, all of which were calculated using QIIME2. Principal coordinates analysis (PCoA), non-metric multidimensional scaling (NMDS), and analysis of similarities (ANOSIM), which reflect the beta diversity of the samples, were calculated using QIIME2, and the corresponding graphs were drawn using the R package.

### 2.6. Statistical analysis

Statistical analysis was performed using SPSS 26.0 software (IBM, Inc., Armonk, NY, United States), and graphs were produced using GraphPad 9.4.0 Software (San Diego, CA, United States). The Kolmogorov–Smirnov test was performed for all continuous data to check for normality. Normally distributed continuous data are presented as mean ± standard deviation (SD), whereas non-normally distributed continuous data are presented as median [interquartile ranges (IQR)]. The student’s t-test, Mann–Whitney U-test, or Kruskal–Wallis test were used for group comparisons as appropriate. Categorical data are presented as counts (percentages) and compared using the Chi-squared test. Survival analysis was performed with the log-rank tests, and the results are presented as Kaplan–Meier curves. Welch’s t-test was applied to identify significant differences in relative taxa abundances using statistical analysis of metagenomic profiles (v2.1.3), and linear discriminant analysis effect size (LEfSe) analysis was used to make high-dimensional non-parametrical comparisons with the cut-off value of linear discriminant analysis score set to 4.0 to identify discriminatory taxa for each group. The correlation between clinical indicators and microorganisms was analyzed using Spearman correlation and displayed via a heatmap. Statistical significance was set at *p* < 0.05.

## 3. Results

### 3.1. The characteristics of patients with SALD

**Figure 1:**
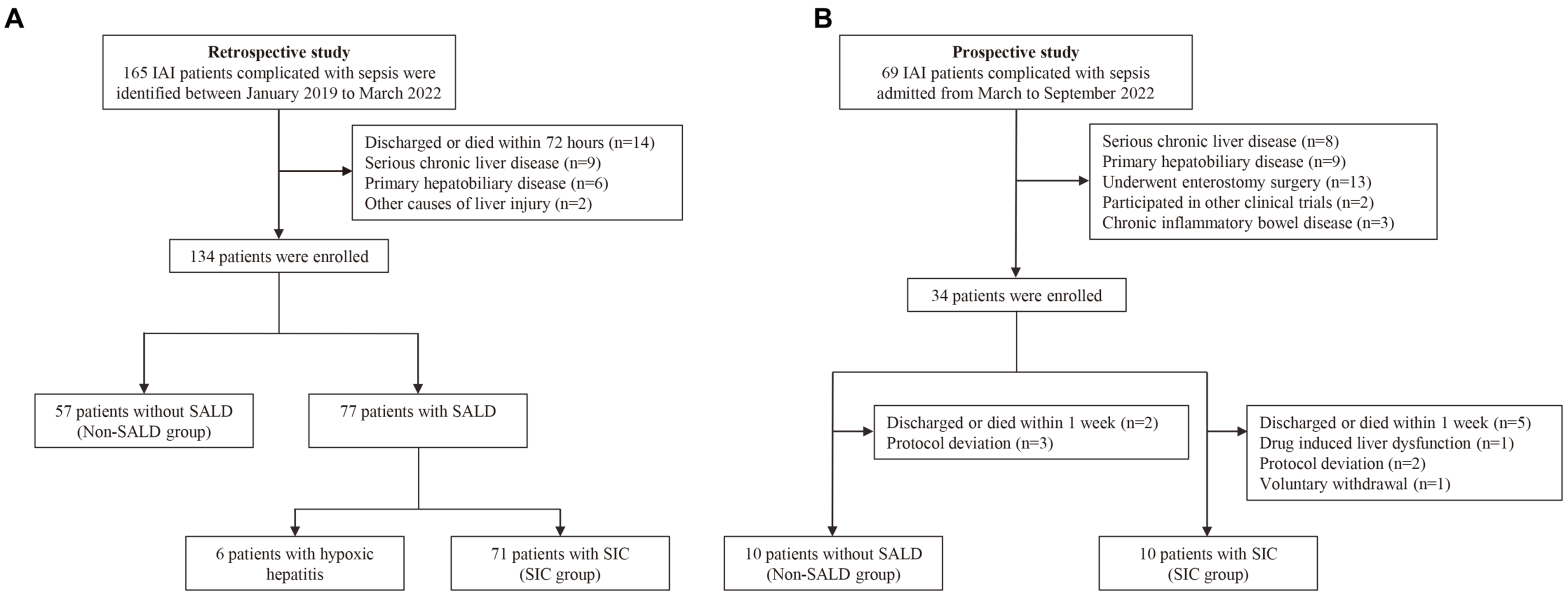
Flow chart of the study. **(A)** The flow chart of patients in the retrospective study. **(B)** The flow chart of patients in the prospective study. IAI, intra-abdominal infection; SALD, sepsis-associated liver dysfunction; *SIC*, sepsis-induced cholestasis.

**Figure 2:**
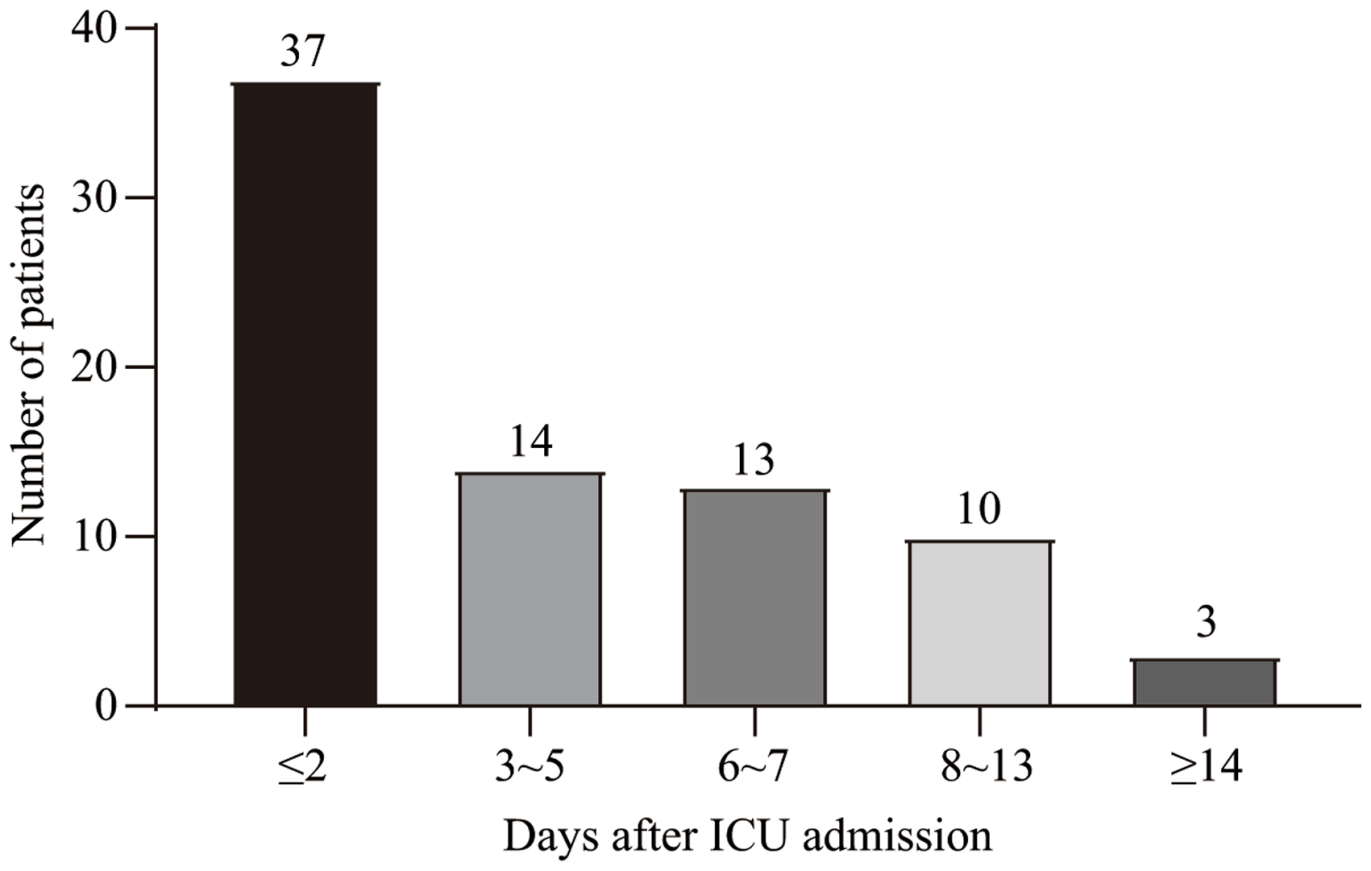
Time distribution of SALD onset in patients with IAI after ICU admission. SALD, sepsis-associated liver dysfunction; IAI, intra-abdominal infection; ICU, intensive care unit.

This study was conducted over three years and included 134 consecutive patients. Seventy-seven patients were diagnosed with SALD, accounting for 57.46%. Figure 1A depicts the participant flow diagram. SALD onset occurred within 2 days of ICU admission in 37 out of 77 patients (48.05%) and on days 3–5 after admission in 14 (18.18%), days 6–7 in 13 (16.88%), days 8–13 in 10 (12.99%), and on day 14 or later in 3 (3.90%) (Figure 2). There were 71 patients with *SIC*, accounting for 92.21% of the patients with SALD. Admission and peak values of serum ALT, AST, TBIL, direct bilirubin, and 𝛾-glutamyl transpeptidase in patients with hypoxic hepatitis and *SIC* are shown in Supplementary Table S1. For patients with hypoxic hepatitis, serum ALT and AST increased rapidly and peaked during the first 72 h after admission (Supplementary Figures S1A,B). Up to 59.15% of patients with *SIC* had increased TBIL on the first day of ICU admission, which lasted for 7–14 days in 49.30% of patients (Supplementary Figure S1C).

### 3.2. Comparison of the general characteristics in the non-SALD and *SIC* groups

As hypoxic hepatitis and *SIC* had different pathological processes and *SIC* was the main characteristic of SALD in our patients, a comparison of clinical characteristics between patients without SALD and with *SIC* was further performed. Table 1 presents the characteristics of the study population. Compared with patients without SALD, those with *SIC* had a lower mean age and longer median ICU LOS (age: 63.87 years vs. 57.45 years, ICU LOS: 7 days vs. 16 days, respectively; all *p* < 0.05) (Table 1). However, no significant difference was observed in the proportion of male, mean APACHE II, and median SOFA scores between the two groups (male: 61.40% vs. 71.83%, APACHE II: 19.01 vs. 20.94, SOFA: 5 vs. 7, respectively; all *p* > 0.05), as well as comorbidity or infection sources.

**Table 1:** Comparison of patient characteristics between non-SALD and SIC groups.

Indicators	All (*n* = 128)	Non-SALD group (*n* = 57)	SIC group (*n* = 71)	*p*-value
Age, mean ± SD, years	60.31 ± 17.20	63.87 ± 17.19	57.45 ± 16.79	0.036
Male, *n* (%)	86 (67.19)	35 (61.40)	51 (71.83)	0.212
APACHE II score, mean ± SD	20.09 ± 8.13	19.01 ± 8.68	20.94 ± 7.61	0.184
SOFA score, median (IQR)	6 (3–10)	5 (3–10)	7 (4–10)	0.219
Comorbidity				
Chronic obstructive pulmonary disease, *n* (%)	29 (22.66)	12 (21.05)	17 (23.94)	0.698
Coronary heart disease, *n* (%)	17 (13.28)	11 (19.30)	6 (8.45)	0.072
Hypertension, *n* (%)	46 (35.94)	24 (42.11)	22 (30.99)	0.193
Diabetes, *n* (%)	20 (15.63)	6 (10.53)	14 (19.72)	0.155
Other, *n* (%)	45 (35.16)	21 (36.84)	24 (33.80)	0.720
Infection site				0.345
Upper gastrointestinal tract, *n* (%)	45 (35.16)	23 (40.35)	22 (30.99)	
Lower gastrointestinal tract, *n* (%)	29 (22.66)	15 (26.32)	14 (19.72)	
Pancreas, *n* (%)	25 (19.53)	9 (15.79)	16 (22.54)	
Other, *n* (%)	29 (22.66)	10 (17.54)	19 (26.76)	
ICU LOS, median (IQR), days	10 (4–29)	7 (3–21)	16 (6–40)	0.005
ICU mortality, *n* (%)	29 (22.66)	8 (14.04)	21 (29.58)	0.037
60-day mortality, *n* (%)	42 (32.81)	11 (19.30)	31 (43.66)	0.004

SALD, sepsis-associated liver dysfunction; *SIC*, sepsis-induced cholestasis; APACHE II, acute physiology and chronic health evaluation II; SOFA, sequential organ failure assessment; ICU, intensive care unit; LOS, length of stay; SD, standard deviation; IQR, interquartile range.

**Figure 3:**
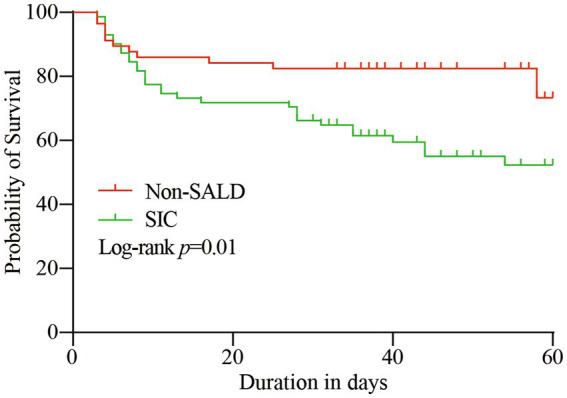
Kaplan–Meier survival curve of the study population. SALD, sepsis-associated liver dysfunction; *SIC*, sepsis-induced cholestasis.

ICU mortality and 60-day mortality in the *SIC* group were significantly higher than the respective mortality rates in the non-SALD group (ICU mortality: 14.04% vs. 29.58%, 60-day mortality: 19.30% vs. 43.66%, respectively; all *p* < 0.05) (Table 1). The Kaplan–Meier method was used to assess mortality with time; a significant difference was observed in the 60-day mortality between the *SIC* and non-SALD groups (*p* < 0.05) (Figure 3).

### 3.3. Comparison of laboratory indicators, complications, and received treatment in the non-SALD and *SIC* groups

Patients in the *SIC* group had significantly higher median WBC count and median neutrophil count, and lower median platelet and mean albumin levels than those in the non-SALD group on ICU admission (WBC count: 7.80 × 10^3^/μL vs. 10.50 × 10^3^/μL, neutrophil count: 6.99 × 10^3^/μL vs. 9.51 × 10^3^/μL, platelet: 158 × 10^3^/μL vs. 121 × 10^3^/μL, albumin: 30.78 g/L vs. 28.76 g/L, respectively; all *p* < 0.05) (Table 2). Moreover, compared with patients in the non-SALD group, patients with *SIC* had a higher incidence of septic shock (49.12% vs. 76.06%, *p* < 0.05), and a greater proportion of these patients required PN for a longer period of time (PN proportion: 15.79% vs. 32.39%, PN duration: 4 days vs. 17 days, respectively; all *p* < 0.05). The incidence of *Acinetobacter baumannii* infection was significantly higher in the *SIC* group than in the non-SALD group (8.77% vs. 22.54%, *p* < 0.05). However, there was no difference between the groups in median lymphocyte count, median monocyte count, mean CRP, median procalcitonin, and median HLA-DR (lymphocyte count: 0.67 × 10^3^/μL vs. 0.57 × 10^3^/μL, monocyte count: 0.30 × 10^3^/μL vs. 0.40 × 10^3^/μL, CRP: 146.42 mg/L vs. 142.65 ± 85.51 mg/L, procalcitonin: 4.58 ng/mL vs. 4.91 ng/mL, HLA-DR: 37.30% vs. 30.40%, respectively; all *p* > 0.05) (Table 2).

**Table 2:** Laboratory indicators, complications, pathogens and treatment information between non-SALD and *SIC* groups during ICU stay.

Indicators	All (*n* = 128)	Non-SALD group (*n* = 57)	*SIC* group (*n* = 71)	*p*-value
Laboratory indicators on admission				
WBC count, median (IQR), 10^3^/μL	9.45 (6.00–14.30)	7.80 (4.65–13.05)	10.50 (6.90–15.70)	0.029
Neutrophil count, median (IQR), 10^3^/μL	8.16 (4.63–12.46)	6.99 (3.50–11.32)	9.51(5.92–13.82)	0.018
Lymphocyte count, median (IQR), 10^3^/μL	0.62 (0.39–0.91)	0.67 (0.44–0.86)	0.57 (0.38–1.08)	0.863
Monocyte count, median (IQR), 10^3^/μL	0.33 (0.19–0.62)	0.30 (0.18–0.59)	0.40 (0.19–0.65)	0.148
Platelet, median (IQR), 10^3^/μL	141 (98–193)	158 (120–222)	121 (89–172)	0.011
C-reactive protein, mean ± SD, mg/L	144.33 ± 83.17	146.42 ± 80.87	142.65 ± 85.51	0.800
Procalcitonin, median (IQR), ng/mL	4.60 (1.23–19.55)	4.58 (0.84–21.35)	4.91 (1.58–18.77)	0.422
HLA-DR, median (IQR), %	35.10 (19.80–56.70)	37.30 (23.13–64.20)	30.40 (19.45–54.30)	0.280
Albumin, mean ± SD, g/L	29.66 ± 5.39	30.78 ± 5.19	28.76 ± 5.41	0.034
Complications				
Respiratory failure, *n* (%)	62 (48.44)	23 (40.35)	39 (54.93)	0.101
Septic shock, *n* (%)	84 (65.63)	28 (49.12)	54 (76.06)	0.001
AKI				0.879
Stage 1, *n* (%)	34 (26.56)	13 (22.81)	21 (29.58)	
Stage 2, *n* (%)	18 (14.06)	6 (10.53)	12 (16.90)	
Stage 3, *n* (%)	16 (12.50)	6 (10.53)	10 (14.08)	
AGI				0.005
Grade I, *n* (%)	16 (12.50)	8 (14.04)	8 (11.27)	
Grade II, *n* (%)	13 (10.16)	3 (5.26)	10 (14.08)	
Grade III, *n* (%)	18 (14.06)	4 (7.02)	14 (19.72)	
Grade IV, *n* (%)	9 (7.03)	1 (1.75)	8 (11.27)	
Bacteremia, *n* (%)	54 (42.19)	21 (36.84)	33 (46.48)	0.286
Treatment received				
Mechanical ventilation, *n* (%)	35 (27.34)	12 (21.05)	23 (32.39)	0.152
Vasopressor therapy≥48 h, *n* (%)	43 (33.59)	14 (24.56)	29 (40.85)	0.053
Renal replacement therapy, *n* (%)	26 (20.31)	11 (19.30)	15 (21.13)	0.798
PN, *n* (%)	32 (25.00)	9 (15.79)	23 (32.39)	0.031
PN duration, median (IQR), days	11.50 (6.25–27.50)	4.00 (4.00–12.50)	17.00 (10.00–38.00)	0.003
Pathogens of ascites				
*Klebsiella pneumoniae*, *n* (%)	29 (22.66)	10 (17.54)	19 (26.76)	0.216
*Acinetobacter baumannii*, *n* (%)	21 (16.41)	5 (8.77)	16 (22.54)	0.037
*Escherichia coli*, *n* (%)	17 (13.28)	7 (12.28)	10 (14.08)	0.765
*Pseudomonas aeruginosa*, *n* (%)	19 (14.84)	8 (14.04)	11 (15.49)	0.818
*Enterococcus faecalis*, *n* (%)	15 (11.72)	6 (10.53)	9 (12.68)	0.707
Antibiotics used				
Against gram-positive bacteria, *n* (%)	51 (39.84)	21 (36.84)	30 (42.25)	0.534
Against gram-negative bacteria, *n* (%)	128 (100)	57 (100)	71 (100)	NA
Against fungi, *n* (%)	22 (17.19)	7 (12.28)	15 (21.13)	0.187

SALD, sepsis-associated liver dysfunction; *SIC*, sepsis-induced cholestasis; ICU, intensive care unit; WBC, white blood cells count; HLA-DR, human leukocyte antigen-DR; AKI, acute kidney injury; AGI, acute gastrointestinal injury; PN, parenteral nutrition; SD, standard deviation; IQR, interquartile rang; NA, no applicable.

### 3.4. Relationship between AGI and *SIC*

**Figure 4:**
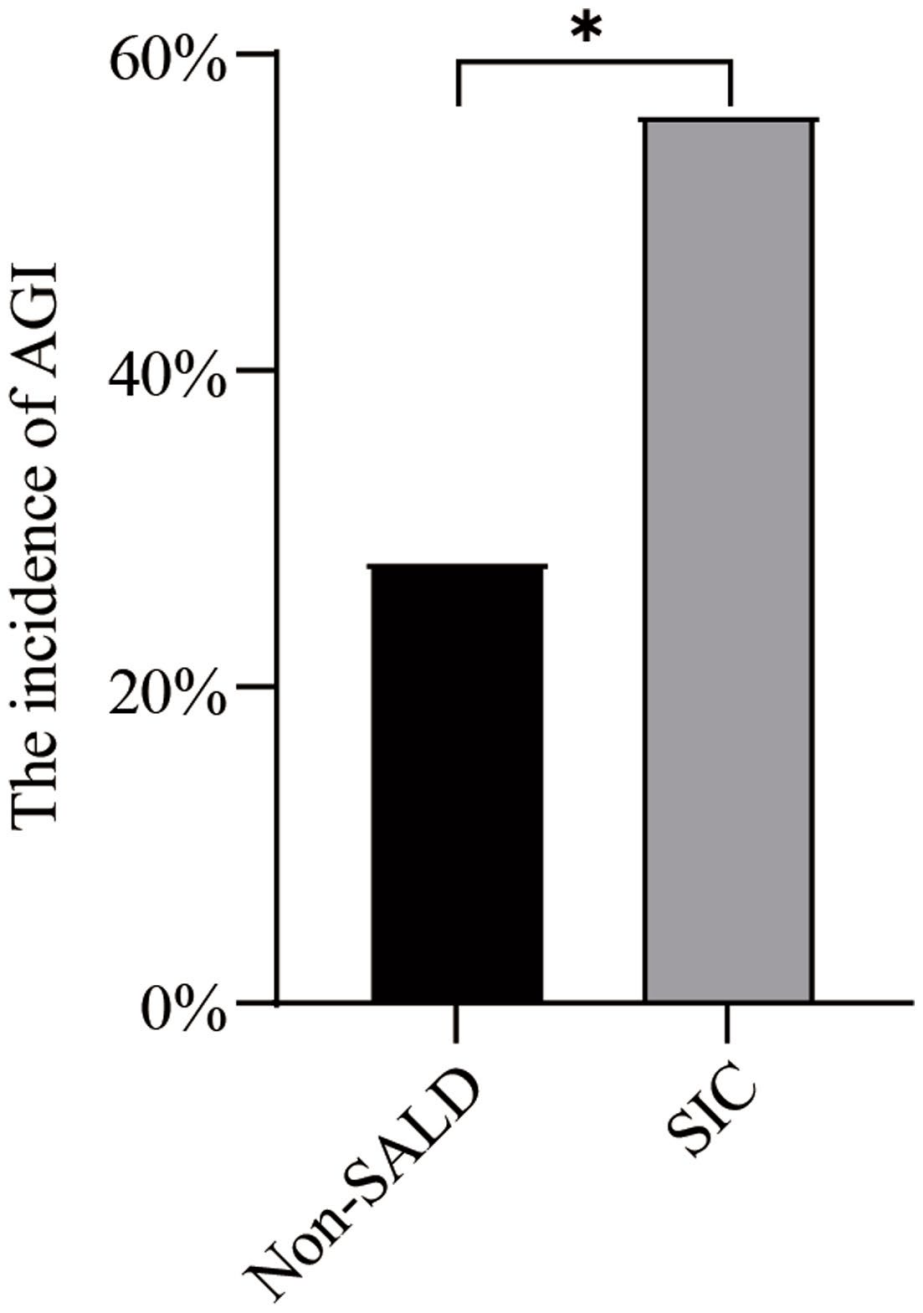
The incidence of AGI in non-SALD and *SIC* groups. AGI, acute gastrointestinal injury; SALD, sepsis-associated liver dysfunction; *SIC*, sepsis-induced cholestasis; *p < 0.05.

**Figure 5:**
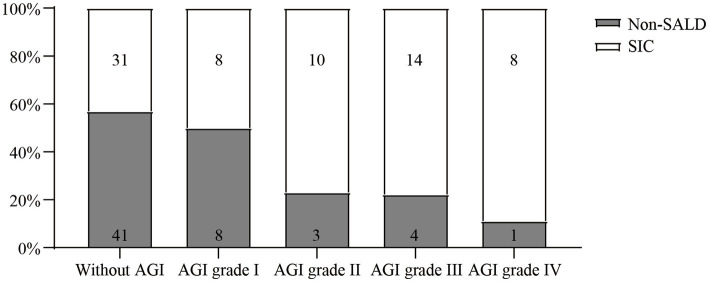
The incidence of *SIC* among IAI patients without AGI and with different AGI grades. *SIC*, sepsis-induced cholestasis; IAI, intra-abdominal infection; AGI, acute gastrointestinal injury.

As shown in Table 2 and Figure 4, Patients with *SIC* had a higher incidence of AGI compared to non-SALD patients (28.07% vs. 56.34%, *p* ≤ 0.05). Furthermore, there were differences in *SIC* incidence according to the grade of AGI in patients with IAI. A severity-dependent relationship was observed with *SIC* occurring in 43.06% of patients without AGI, 50.00% of patients with AGI grade I, 76.92% of patients with AGI grade II, 77.78% of patients with AGI grade III, and 88.89% of patients with AGI grade IV (Figure 5).

### 3.5. Gut microbiota features of non-SALD and *SIC* individuals

Figure 1B describes the process for selecting study participants. Twenty patients with IAI were enrolled for gut microbiota analysis during the 6-month study period. Supplementary Table S2 presents the basic characteristics of the patients in non-SALD and *SIC* groups. No significant difference was observed in age, sex, APACHE II score, SOFA score, comorbidity, and infection source between the two groups (all *p* > 0.05). We collected 60 fecal samples for analysis from 10 patients each in the non-SALD and *SIC* groups on days 1, 3, and 7 after ICU admission. Finally, 4,578,880 high-quality sequences were obtained from the fecal samples after quality control procedures (Supplementary Figure S2).

### 3.6. Comparison of gut microbiota composition between non-SALD and *SIC* groups on day 1 after ICU admission

**Figure 6:**
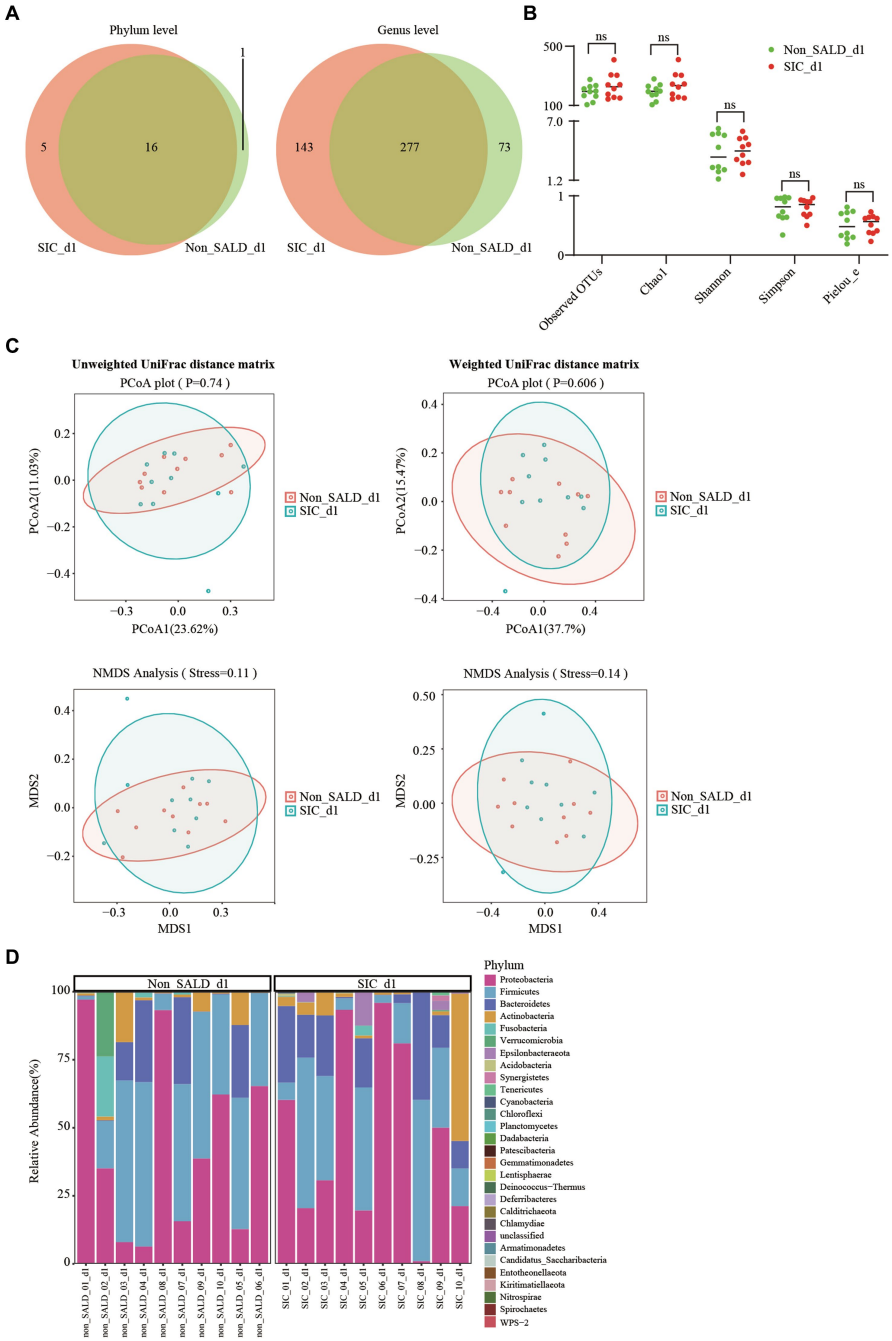
Gut microbiota of patients with IAI without SALD or with *SIC* on day 1 after ICU admission. **(A)** Venn diagram of the gut microbiota in the non-SALD and *SIC* groups at the phylum and genus levels, respectively. **(B)** Alpha diversity differences between non-SALD and *SIC* groups were estimated using the observed OTUs, Chao1, Shannon, Simpson, and Pielou_e indices. **(C)** Beta diversity differences between non-SALD and *SIC* groups were estimated using the PCoA plot and NMDS analysis based on unweighted and weighted UniFrac distance matrixes. **(D)** Gut microbiota composition at the phylum level among non-SALD and *SIC* groups. IAI, intra-abdominal infection; SALD, sepsis-associated liver dysfunction; *SIC*, sepsis-induced cholestasis; ICU, intensive care unit; OTU, operational taxonomic units; PCoA, principal coordinates analysis; NMDS, non-metric multidimensional scaling; non_SALD_d1 represents day 1 in the non-SALD group; *SIC*_d1 represents day 1 in *SIC* group; non_SALD_01_d1 represents day 1 for patient 01 in the non-SALD group; *SIC*_01_d1 represents day 1 for patient 01 in *SIC* group; ns, *p* ≥ 0.05.

On day 1 after ICU admission, Venn diagram analysis indicated that 1 phylum and 73 genera were present in the non-SALD group, and 5 phyla and 143 genera were present in the *SIC* group (Figure 6A). No significant difference was observed in the indicators related to alpha diversity between the two groups, including observed OTUs, Chao1, Shannon, Simpson, and Pielou_e (observed OTUs: 195 vs. 226, Chao1: 195.47 vs. 234.17, Shannon: 3.48 vs. 4.07, Simpson: 0.82 vs. 0.86, Pielou_e: 0.48 vs. 0.57, respectively; all *p*>0.05) (Figure 6B; Supplementary Table S3). PCoA, NMDS, and ANOSIM based on unweighted and weighted UniFrac distance matrixes revealed that there were no significant differences in the gut microbiota composition between the two groups (all *p* > 0.05) (Figure 6C; Supplementary Figure S3). At the phylum level, no significant difference was observed in the gut microbiota composition between the two groups. *Proteobacteria* and *Firmicutes* were the dominant phyla, accounting for 43.46% and 36.80% of the total bacteria in the non-SALD group and 47.37% and 26.95% in the *SIC* group, respectively (Figures 4, 6D; Supplementary Table S3).

### 3.7. Comparison of gut microbiota composition between non-SALD and *SIC* groups on day 7 after ICU admission

**Figure 7:**
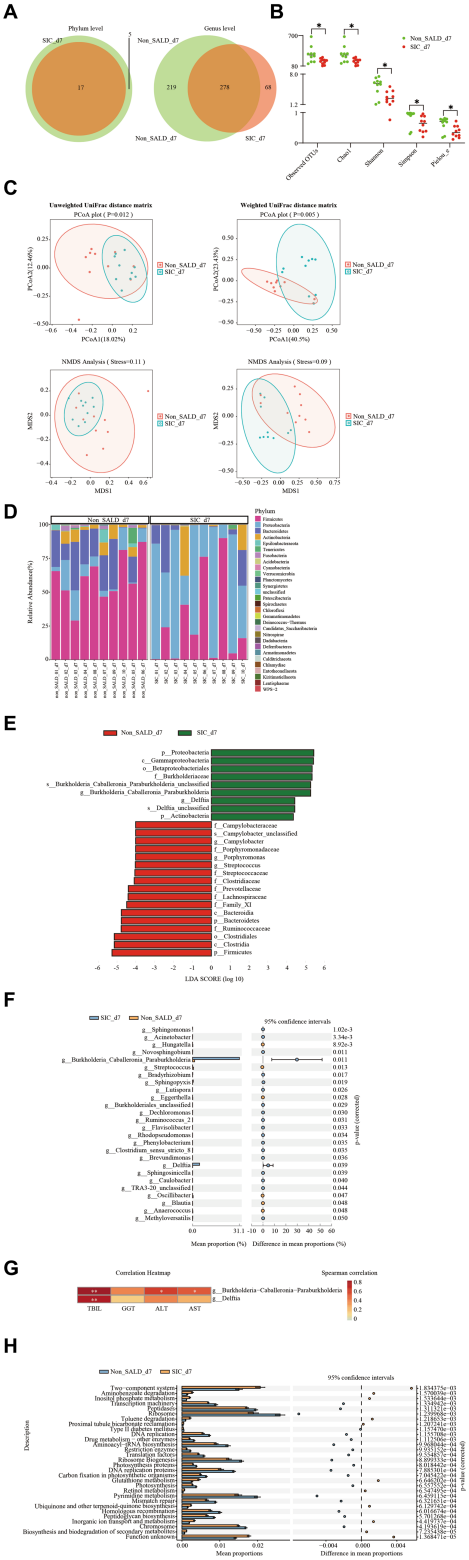
Gut microbiota of patients with IAI without SALD or with *SIC* on day 7 after ICU admission. **(A)** Venn diagram of the gut microbiota in the non-SALD and *SIC* groups at the phylum and genus levels, respectively. FIGURE 7 (Continued)**(B)** Alpha diversity differences between non-SALD and *SIC* groups were estimated using the observed OTUs, Chao1, Shannon, Simpson, and Pielou_e indices. **(C)** Beta diversity differences between non-SALD and *SIC* groups were estimated using the PCoA plot and NMDS analysis based on unweighted and weighted UniFrac distance matrixes. **(D)** Gut microbiota composition at the phylum level among non-SALD and *SIC* groups. **(E)** Histogram of LDA scores for differentially abundant taxa between non-SALD and *SIC* groups. Only taxa with an LDA > 4 are shown. **(F)** Relative abundances of 22 differentially expressed genera taxa were evaluated using STAMP. The left and right panels demonstrated the average relative abundance and the 95% confidence interval of each genus in the non-SALD and *SIC* groups, respectively. **(G)** Spearman correlation heatmap of the relationship between the two significantly different bacterial genera and liver function indicators. **(H)** Gut microbiota functions against KEGG database between the non-SALD and *SIC* groups predicted by PICRUSt analysis. IAI, intra-abdominal infections; SALD, sepsis-associated liver dysfunction; *SIC*, sepsis-induced cholestasis; ICU, intensive care unit; OTU, operational taxonomic units; PCoA, principal coordinates analysis; NMDS, non-metric multidimensional scaling; LDA, linear discriminant analysis; STAMP, statistical analysis of metagenomic profiles; PICRUSt, the phylogenetic investigation of communities by reconstruction of unobserved states; non_SALD_d7 represents day 7 in the non-SALD group; *SIC*_d7 represents day 7 in *SIC* group; non_SALD_01_d7 represents day 7 for patient 01 in the non-SALD group; *SIC*_01_d7 represents day 7 for patient 01 in *SIC* group; *p < 0.05; **p < 0.001.

On day 7, Venn diagram analysis indicated that 5 phyla and 219 genera were present in the non-SALD group only, whereas 68 genera were present in the *SIC* group (Figure 7A). Non-SALD participants had markedly higher median observed OTUs, median Chao1, median Shannon, median Simpson, and median Pielou_e than *SIC* individuals (observed OTUs: 304 vs. 185, Chao1: 305.29 vs. 188.94, Shannon: 5.91 vs. 2.56, Simpson: 0.97 vs. 0.65, Pielou_e: 0.71 vs. 0.34, respectively; all *p* < 0.05) (Figure 7B; Supplementary Table S3). There were also significant differences in the indices of beta diversity, including PCoA, NMDS, and ANOSIM analysis, in the gut microbiota composition between the non-SALD and *SIC* groups (all *p* < 0.05) (Figure 7C; Supplementary Figure S5).

According to the stacked bar plots of the top 30 abundant phylum taxonomic levels, *Firmicutes* was the dominant phyla in the non-SALD group, accounting for 59.88% of the total bacteria. Meanwhile, *Proteobacteria* was the most abundant phylum in the gut microbiota in the *SIC* group, accounting for 57.89% (Figure 7D; Supplementary Table S3). A cladogram was generated using LEfSe to identify the specific bacteria associated with *SIC* (Supplementary Figure S6). LEfSe analysis revealed a clear alteration of the gut microbiota composition characterized by higher phylum *Firmicutes* and *Bacteroidetes* in the non-SALD group and higher phylum *Proteobacteria* and *Actinobacteria* in the *SIC* group (Figure 7E). There were significant differences in the relative abundances of these phyla taxa between the two groups (*Firmicutes*: 59.88% vs. 27.36%, *Bacteroidetes*: 20.43% vs. 8.32%, *Proteobacteria*: 9.66% vs. 57.89%, *Actinobacteria*: 5.47% vs. 5.77%, respectively; all *p* < 0.05) (Supplementary Table S3; Figure 7).

Furthermore, 22 significantly different genera taxa among the two groups were screened using statistical analysis of metagenomic profiles and random forests with the genus-level relative abundance data created to investigate which taxa contributed to the observed differences (Figure 7F; Supplementary Table S3). More importantly, *Burkholderia-Caballeronia-Paraburkholderia* and *Delftia* were the most dominant and the second most dominant genera, respectively (*Burkholderia-Caballeronia-Paraburkholderia*: 1.4 vs. 31.13, *Delftia*: 0.04 vs. 4.60, respectively; all *p* < 0.05). Sankey plots revealed the pairing interactions between the phylum and genus levels in patients with non-SALD and *SIC* (Supplementary Figure S8). Gut microbiotas in the non-SALD and *SIC* groups were mainly *Proteobacteria* and *Firmicutes*, of which *Burkholderia-Caballeronia-Paraburkholderia* and *Enterococcus* were the two most abundant genera. Additionally, Spearman’s correlations between liver functional indicators and the abundance of *Burkholderia-Caballeronia-Paraburkholderia* and *Delftia* were calculated. The abundance of both taxa had a strong positive correlation with the TBIL level (all *p* < 0.001). Furthermore, the abundance of *Burkholderia − Caballeronia − Paraburkholderia* had a significant but weak correlation with AST and ALT levels, respectively (all *p* < 0.05) (Figure 7G).

The phylogenetic investigation of communities by reconstruction of unobserved states (PICRUSt) analysis was performed to infer the potential function of gut microbiota. The results showed significant differences in 256 KEGG pathways between the non-SALD and *SIC* groups. The top 30 pathways with the most significant differences are illustrated in Figure 7H, including 19 pathways enriched in the non-SALD group and 11 enriched in the *SIC* group. Ribosome, pyrimidine metabolism, and the two-component system were the top three pathways with the most significant differences, which may play important roles during the process of *SIC*.

### 3.8. Dynamic changes of gut microbiota composition in the non-SALD and *SIC* groups during the first week after ICU admission

**Figure 8:**
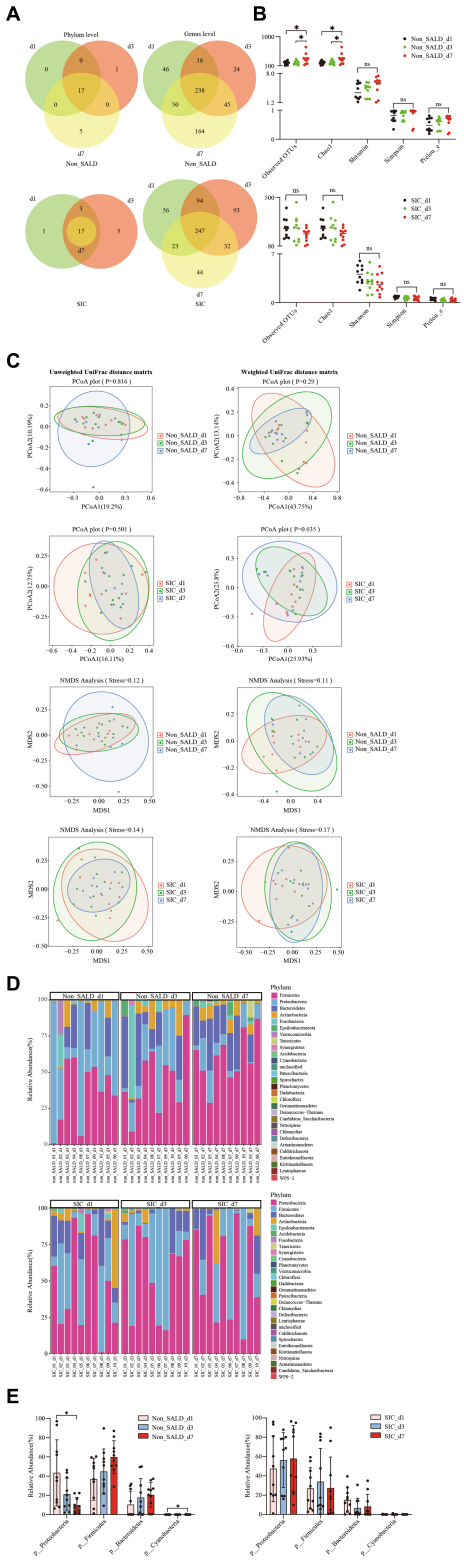
Dynamic changes of gut microbiota in patients with IAI without SALD or with *SIC* during the first week. **(A)** Venn diagram of the gut microbiota on days 1, 3, and 7 after ICU admission in the non-SALDFIGURE 8 (Continued)and *SIC* groups at the phylum and genus levels, respectively. **(B)** Dynamic changes of alpha diversity on days 1, 3, and 7 after ICU admission were estimated using the observed OTUs, Chao1, Shannon, Simpson, and Pielou_e indices in the non-SALD and *SIC* groups, respectively. **(C)** Beta diversity differences on days 1, 3, and 7 after ICU admission were estimated using the PCoA plot and NMDS analysis based on unweighted and weighted UniFrac distance matrixes in the non-SALD and *SIC* groups, respectively. **(D)** Gut microbiota composition on days 1, 3, and 7 after ICU admission at the phylum level in the non-SALD and *SIC* groups. **(E)** Comparison of the gut microbiota composition at the phylum level at days 1, 3, and 7 after ICU admission in the non-SALD and *SIC* groups, respectively. IAI, intra-abdominal infection; SALD, sepsis-associated liver dysfunction; *SIC*, sepsis-induced cholestasis; ICU, intensive care unit; OTU, operational taxonomic units; PCoA, principal coordinates analysis; NMDS, non-metric multidimensional scaling; non_SALD_d1 represents day 1 in the non-SALD group; non_SALD_d3 represents day 3 in the non-SALD group; non_SALD_d7 represents day 7 in the non-SALD group; *SIC*_d1 represents day 1 in the *SIC* group; *SIC*_d3 represents day 3 in the *SIC* group; *SIC*_d7 represents day 7 in *SIC* group; non_SALD_01_d1 represents day 1 for patient 01 in non-SALD group; SIC_01_d1 represents day 1 for patient 01 in the SIC group; ns, *p* ≥ 0.05; *p < 0.05.

Venn diagrams at different time points in the non-SALD group indicated that 46 genera, 1 phylum and 24 genera, and 5 phyla and 164 genera were present on days 1, 3, and 7, respectively. In the *SIC* group, Venn diagrams indicated that 1 phylum and 56 genera, 5 phyla and 93 genera, and 44 genera were present on days 1, 3, and 7 (Figure 8A). Shannon, Simpson, and Pielou_e did not differ in the non-SALD group between days 1, 3, and 7 (Shannon: 3.48 vs. 4.67 and 5.91, Simpson: 0.82 vs. 0.91 and 0.97, Pielou_e: 0.48 vs. 0.62 and 0.71, respectively; all *p* > 0.05). However, significant differences were found in observed OTUs and Chao1 between days 1, 3, and 7(observed OTUs: 195 vs. 180 and 304, Chao1: 195.47 vs. 184.5 and 305.29, respectively; all *p* < 0.05) (Figure 8B). The median observed OTUs, median Chao1, median Shannon, median Simpson, and median Pielou_e had no significant difference on days 1, 3, and 7 in the *SIC* group (observed OTUs: 226 vs. 236 and 185, Chao1: 234.17 vs. 236.61 and 188.94, Shannon: 4.07 vs. 2.99 and 2.56, Simpson: 0.86 vs. 0.63 and 0.65, Pielou_e: 0.57 vs. 0.38 and 0.34, respectively; all *p* > 0.05). Moreover, no difference was observed in the beta diversity, including PCoA, NMDS, and ANOSIM analysis, at different time points in the two groups (all *p* > 0.05) (Figure 8C; Supplementary Figure S9). At the phylum level, the abundance of *Proteobacteria* decreased gradually with *Firmicutes*, *Bacteroidetes,* and *Cyanobacteria* increased gradually from day 1 to day 7 in the non-SALD group (Figure 8D; Supplementary Figure S10). However, only the changes in *Proteobacteria* and *Cyanobacteria* were significant (*Proteobacteria*: 43.46% vs. 21.23% vs. 9.66%, *Cyanobacteria*: 0.01% vs. 0% vs. 0.11%, respectively; all *p* < 0.05) (Figure 8E). On the contrary, there was no significant difference in the abundances of *Proteobacteria*, *Firmicutes,* and *Bacteroidetes* in the *SIC* group (Figure 8E; Supplementary Figure S10).

## 4. Discussion

To the best of our knowledge, this is the first study to explore the relationship between AGI, gut microbiota, and *SIC* in critically ill patients with IAI. Our results demonstrated that the AGI incidence was significantly higher in the *SIC* group compared to the non-SALD group, and a severity-dependent relationship was found between AGI grade and *SIC* occurrence. Furthermore, the study found that patients with IAI-related *SIC* suffer more severe gut microbiota disorders than those without SALD.

In our study, *SIC* was the main clinical feature in 92.21% of patients with IAI-related SALD, consistent with the findings of another study reporting a 96.6% incidence of hyperbilirubinemia (defined as TBIL >2 mg/dL) (7). This suggests that cholestasis is the most common gastrointestinal sign of SALD in patients with IAI. Liver histopathology also showed intrahepatic cholestasis, with few or no hepatocyte necroses in patients with extrahepatic origin infection (17). Moreover, our study found that patients with *SIC* had a long duration of elevated TBIL, approximately 7–14 days.

Primary abdominal diseases, such as peritonitis, abdominal surgery, or trauma, usually lead to a more severe AGI than other diseases that indirectly affect the gastrointestinal system (9). A prospective study by Derikx JP et al. revealed that on admission, nonsurvivors with IAI had higher plasma fatty acid binding protein levels, a marker of cellular damage, in the gut and liver than nonsurvivors with pneumonia (18). In addition, IAI is often complicated by intra-abdominal hypertension (IAH), further aggravating intestinal damage (19). In a rabbit model, IAI combined with IAH could further aggravate intestinal mucosal tissue damage and permeability and alter the intestinal mucosa redox status.

By comparing the gut microbiota composition of the non-SALD and *SIC* groups in patients with IAI, some significant changes were observed in the abundance, distribution, and structure of the gut microbiota under *SIC* conditions. Among the two most dominant phyla taxa, the abundance of *Proteobacteria* in the *SIC* group increased gradually during the first week after ICU admission, and the abundance of *Firmicutes* did not alter, while the abundance of *Proteobacteria* in the non-SALD group decreased and that of *Firmicutes* increased gradually; there were significant differences in both taxa on day 7 between the two groups. The abundance of *Firmicutes* improved in the non-SALD group, although this was lower than the approximately 80% abundance reported in healthy individuals. Many gram-negative bacteria in the phylum *Proteobacteria* are pathogenic and lipopolysaccharide-containing (20, 21), such as *Escherichia coli* and *Klebsiella*. This suggests that patients with *SIC* may suffer from more severe gut inflammation than patients with non-SALD because gram-negative bacteria can produce numerous lipopolysaccharides, some of which are powerful endotoxins. Moreover, gut microbiota can produce bacteriocins to protect against invading pathogens, and bacteriocins are mainly produced by *Firmicutes* (22). Moreover, some specific members of *Firmicutes*, such as genera *Clostridium* and *Bacillus,* are in vegetative growth or spore state. The ability to produce spores has ecological advantages for the organism because it enables it to survive in adverse conditions, thus, efficiently colonizing the gut (20). In a prospective study involving gut microbiota analysis of 34 critically ill patients and 15 healthy volunteers, Lankelma JM et al. Discovered that critically ill patients had a significantly higher abundance of *Proteobacteria* and a lower abundance of *Firmicutes* and *Bacteroidetes* than healthy volunteers (23). Similarly, Yang XJ et al. Prospectively analyzed 10 patients with sepsis, 10 non-septic patients, and 10 healthy individuals. They discovered that *Firmicutes* was the dominant phylum in patients with or without sepsis on day 1 after ICU admission (24). Compared with healthy individuals, *Bacteroidetes* significantly decreased in patients with or without sepsis, while *Proteobacteria* increased, although there was no significant difference between the two groups. Moreover, the gut microbiota composition of patients with sepsis did not improve significantly within the first week (24).

Several reasons may explain the disparities in gut microbiota composition observed in our study. First, in the case of sepsis, hypoperfusion, and ischemia or reperfusion insults cause intestinal mucosal injury, which manifests as intestinal mucosal inflammation and a series of changes in the microenvironment, including increased nitrate concentration and altered mucosal oxygen gradient (25, 26). These changes are more conducive to *Proteobacteria* growth, including many pathogenic gram-negative bacteria, such as *Pseudomonas aeruginosa* and *E. coli*. This is also supported by the fact that a higher proportion of patients with septic shock were observed in the *SIC* group than in the non-SALD group in our study. Second, the liver is critical in regulating immune and inflammatory responses. During sepsis, Kupffer cells, macrophages, and other immune cells are activated, producing numerous inflammatory mediators, including interferons, interleukin-6, interleukin-1β, interleukin-8, and tumor necrosis factor (14, 27). These directly affect the protein expression of claudins, junctional adhesion molecule A, occludin, and zonula occludens-1, thus, damaging the intestinal barrier and increasing gut permeability (2). Moreover, the liver also secretes bile acids and antimicrobial molecules, which are transported to the gut through the hepato-intestinal circulation to maintain the gut microbiome homeostasis. Under *SIC* conditions, this homeostasis is disrupted, resulting in the disruption of the gut microbiota. Third, altered hormone levels, proton pump inhibitors, nutritional support, and broad-spectrum antibiotics contribute to gut microbiota disorder in critically ill patients (28).

Several animal experiments have confirmed that gut microbiota disorder plays an important role in the onset and progression of liver injury in the sepsis model. In the cecal ligation and puncture-induced septic mice model, the abundance of phylum *Firmicutes* and *Actinobacteria* in sepsis-sensitive mice, defined as a survival time of approximately 24 h after cecal ligation and puncture, was lower than that in sepsis-resistant mice, defined as a survival time of 7 days. However, there was no significant difference in *Firmicutes* (12). Moreover, mice received fecal microbiota transplantation from the feces of sepsis-sensitive mice and developed more severe liver dysfunction when compared with that of sepsis-resistant mice. Another study by Liu Z et al. discovered that the abundance of *Proteobacteria, Bacteroidetes,* and *Actinobacteria* increased significantly, whereas that of *Firmicutes* decreased significantly in the gut microbiota of patients with sepsis vs. healthy individuals (29). Subsequently, the cecal ligation and puncture-treated mice received septic feces and exhibited more severe liver inflammation and damage than those receiving healthy feces. The unique anatomical location of the liver is the basis for the liver-gut axis, which transports microbial metabolites and nutrients to the liver through the portal vein or arterial blood to maintain healthy liver metabolism (30).

At the genus level, in addition to a decline in the abundance of beneficial symbiotic bacteria such as *Lactobacillus* and *Oscillibacter*, other opportunistic pathogens, including *Rothia* and *Hungatella*, also declined in patients with *SIC*. One possible explanation for this phenomenon is that a higher percentage of patients in the *SIC* group developed AGI and were more likely to receive selective digestive decontamination, which mainly refers to the administration of non-absorbable antibiotics, such as polymyxin and vancomycin, through the gastrointestinal tract (31, 32). The purpose of selective digestive decontamination is to preserve beneficial symbiotic bacteria while preventing the colonization of opportunistic pathogens. However, this is a controversial approach because the effect on gut microbiota composition remains unclear in critically ill patients. *Lactobacillus* is a dominant bacterium in the gut that offers various health benefits to the host, including an increase in short-chain fatty acid levels in the feces and up-regulating the body’s immune response (33). *Oscillibacter,* as an anti-inflammatory bacterium, regulates T cell differentiation by reducing Th17 polarization, which secretes IL-17 cytokine, and promoting and maintaining the IL-10-producing Treg cells in the gut of a hepatocellular carcinoma mouse model (34). Some studies have discovered that a decline in the abundance of *Oscillibacter* in the gut is associated with obesity and type 2 diabetes (35, 36). *Rothia* plays an important role in the pathogenesis of pneumonia and bacteremia, particularly in immunocompromised patients or those with indwelling intravascular foreign bodies (37). *Hungatella* is an anaerobic bacterium isolated from feces, and one of its species, *hathewayi,* has been identified in patients with bacteremia and liver abscesses (38). Furthermore, an increased abundance of *Hungatella* in the gut microbiota has been associated with Parkinson’s disease (39).

Among the 22 genera taxa with significant differences between the two groups, the abundance of *Burkholderia − Caballeronia − Paraburkholderia* and *Delftia* increased the most in the *SIC* group. As a member of *Proteobacteria, Burkholderia − Caballeronia − Paraburkholderia* is elevated in ulcerative colitis (40). In a mouse model of breast cancer after chemotherapy, the abundance of *Burkholderia − Caballeronia − Paraburkholderia* in gut microbiota was positively correlated with the expression of pro-inflammatory mediators and negatively correlated with the expression of tight junction proteins in gut tissue (41). Moreover, Zhang T et al. reported that *Burkholderia − Caballeronia − Paraburkholderia* combined with *Faecalibacterium* and *Ruminococcus_1* in gut microbiota could be used for the early diagnosis of diagnosing cholangiocarcinoma, which is better than the traditional carbohydrate antigen 19-9 indicator (42). *Delftia* is another member of *Proteobacteria*. Naito T et al. identified a crypt-specific core microbiota in murine cecal and proximal colonic crypts, mainly composed of *Acinetobacter*, *Delftia,* and *Stenotrophomonas* (43). Furthermore, the microbiota was characterized by strict aerobic and non-fermentative bacteria. The crypt-specific core microbiota is important in regulating the balance between proliferation and differentiation in colonic epithelial cells. Additionally, several other investigators reported that the abundance of *Delftia* in gut microbiota increased in some intestinal diseases, such as colorectal cancer and irritable bowel syndrome (44, 45).

Microbial function prediction analysis was also performed in patients with IAI with non-SALD and *SIC*, which showed that ribosome, pyrimidine metabolism, and the two-component system were the top three KEGG pathways with significant differences associated with gut microbiota between the two groups. The two−component system was enriched in the *SIC* group, whereas ribosome and pyrimidine metabolism were depleted. This is consistent with the findings reported by Wu J et al. (46) involving 33 patients with acute hepatitis E and 25 healthy individuals. The authors observed that ribosome and pyrimidine metabolism associated with gut microbiota were enriched in the healthy control group, and the two−component system was enriched in the acute hepatitis E group.

Our study had some limitations. First, this is a single-center, observational study with a small sample size, particularly the gut microbiota study, leading to a certain degree of selective bias. Second, many endogenous and exogenous factors may influence the gut microbiota in critically ill patients, including age, proton pump inhibitors, nutritional support, and broad-spectrum antibiotics. Therefore, more rigorous, larger sample sizes, and multi-center prospective studies are required in the future to minimize the interference from these factors. Last, our study did not explore the metabolic function of the gut microbiota. More studies are required to confirm the relationship between gut microbial metabolites and *SIC*.

## 5. Conclusion

There is a severity-dependent relationship between AGI grade and *SIC* occurrence in adult patients with IAI. The different gut microbiota compositions were revealed in patients without SALD or with *SIC* during the first week after ICU admission. Patients with *SIC* are characterized by lower microbiota diversities, decreased abundance of *Firmicutes* and *Bacteroidetes,* and increased abundance of *Proteobacteria* and *Actinobacteria* at the phylum level compared to patients without SALD. Furthermore, the *SIC*-associated microbial consortium and marker taxa at the genus level were identified, especially *Burkholderia − Caballeronia − Paraburkholderia* and *Delftia*. These findings may provide essential guidance for the future treatment of *SIC* in patients with IAI.

## Data availability statement

The datasets presented in this study can be found in online repositories. The names of the repository/repositories and accession number(s) can be found in the article/Supplementary material.

## Ethics statement

The studies involving human participants were reviewed and approved by the Medical Ethics Committee of Nanjing Drum Tower Hospital. The patients/participants provided their written informed consent to participate in this study.

## Author contributions

BZ: study concept and design, methodology, investigation, and writing and revising the manuscript. XC: investigation, validation, and writing the original manuscript. CH and TS: collection and assembly of data and writing the original manuscript. KC: verification of data. XL and JD: data analysis and interpretation. MC: English editing. ZZ and WY: study concept and study design, article revision, and article approval. All authors contributed to the article and approved the submitted version.

## Funding

This work was supported by the National Natural Science Foundation of China (number 81927808).

## Conflict of interest

The authors declare that the research was conducted in the absence of any commercial or financial relationships that could be construed as a potential conflict of interest.

## Publisher’s note

All claims expressed in this article are solely those of the authors and do not necessarily represent those of their affiliated organizations, or those of the publisher, the editors and the reviewers. Any product that may be evaluated in this article, or claim that may be made by its manufacturer, is not guaranteed or endorsed by the publisher.

## Glossary

SALD: sepsis-associated liver dysfunction
IAI: intra-abdominal infection
ICU: intensive care unit
AGI: acute gastrointestinal injury
*SIC*: sepsis-induced cholestasis
APACHE: acute physiology and chronic health evaluation
SOFA: sequential organ failure assessment
WBC: white blood cells
CRP: C-reactive protein
HLA-DR: human leukocyte antigen-DR
AKI: acute kidney injury
PN: parenteral nutrition
LOS: length of stay
ALT: alanine aminotransferase
AST: aspartate aminotransferase
TBIL: total bilirubin
OTUs: operational taxonomic units
PCoA: principal coordinates analysis
NMDS: non-metric multidimensional scaling
ANOSIM: analysis of similarities
STAMP: statistical analysis of metagenomic profiles
LDA: linear discriminant analysis
LEfSe: linear discriminant analysis effect size
