# Corrigendum: Acute gastrointestinal injury and altered gut microbiota are related to sepsis-induced cholestasis in patients with intra-abdominal infection: a retrospective and prospective observational study

**DOI:** 10.3389/fmed.2023.1280907

**Published:** 2023-08-29

**Authors:** Beiyuan Zhang, Xiancheng Chen, Chenhang He, Ting Su, Ke Cao, Xiaoyao Li, Jianfeng Duan, Ming Chen, Zhanghua Zhu, Wenkui Yu

**Affiliations:** ^1^Department of Critical Care Medicine, Affiliated Drum Tower Hospital, Medical School of Nanjing University, Nanjing, Jiangsu Province, China; ^2^Nanjing Drum Tower Clinical College of Xu Zhou Medical University, Nanjing, China

**Keywords:** intra-abdominal infection, sepsis-induced cholestasis, acute gastrointestinal injury, gut microbiota, 16S ribosomal ribonucleic acid sequencing

In the published article, there was an error in affiliation 1. Instead of “Nanjing Drum Tower Hospital, Nanjing, Jiangsu Province, China”, it should be “Department of Critical Care Medicine, Affiliated Drum Tower Hospital, Medical School of Nanjing University, Nanjing, Jiangsu Province, China”.

The authors apologize for this error and state that this does not change the scientific conclusions of the article in any way. The original article has been updated.

