# Supplementary material for: Acute gastrointestinal injury and altered gut microbiota are related to sepsis-induced cholestasis in patients with intra-abdominal infection: a retrospective and prospective observational study
Source: Front Med (Lausanne). 2023 Jul 27;10:1144786. doi: 10.3389/fmed.2023.1144786 (PMC10414538; doi:10.3389/fmed.2023.1144786)
Supplement: Supplementary file 1 [file Data_Sheet_1.docx]

Supplementary Material

Acute gastrointestinal injury and altered gut microbiota are related to sepsis-induced cholestasis in patients with intra-abdominal infection: a retrospective and prospective observational study

Beiyuan Zhang†, Xiancheng Chen†, Chenhang He†, Ting Su†, Ke Cao, Xiaoyao Li, Jianfeng Duan, Ming Chen, Zhanghua Zhu, Wenkui Yu^*^

*** Correspondence:**

Professor Wenkui Yu: [yudrnj@163.com](mailto:yudrnj@163.com)

## Supplementary tables

**Supplementary Table 1.** Admission and peak values of liver function indicators in patients with hypoxic hepatitis and SIC

| Indicators | Hypoxic hepatitis  (n =6) | SIC  (n =71) |
| --- | --- | --- |
| Admission ALT, median (IQR), IU/L | 446.05(36.95–840.55) | 25.30(13.20–42.80) |
| Peak ALT, median (IQR), IU/L | 1386.15(825.35–2749.40) | 83.80(43.80–213.90) |
| Admission AST, median (IQR), IU/L | 593.60(43.13–1712.00) | 40.00(24.00–69.00) |
| Peak AST, median (IQR), IU/L | 2547.60(1481.00–8222.68) | 118.00(57.00–300.60) |
| Admission TBIL, median (IQR), mg/dL | 0.67(0.40–1.25) | 2.04(0.95–3.29) |
| Peak TBIL, median (IQR), mg/dL | 1.73(1.39–1.99) | 4.78(2.81–11.36) |
| Admission DBIL, median (IQR), mg/dL | 0.29(0.18–0.66) | 0.98(0.45–2.02) |
| Peak DBIL, median (IQR), mg/dL | 0.98(0.89–1.22) | 2.16(1.39–3.53) |
| Admission GGT, median (IQR), IU/L | 21.55(10.83–69.28) | 32.80(21.25–64.05) |
| Peak GGT, median (IQR), IU/L | 60.00(31.65–202.05) | 122.35(57.00–233.65) |

Abbreviations: SIC, sepsis-induced cholestasis; ALT, alanine aminotransferase; AST, aspartate aminotransferase; TBIL, total bilirubin; DBIL, direct bilirubin; GGT, 𝛾-glutamyl transpeptidase, IQR, interquartile range.

**Supplementary Table 2.** Comparison of basic characteristics between non-SALD and SIC groups

| Indicators | Non-SALD group  (n =10) | SIC group  (n=10) | *p*-value |
| --- | --- | --- | --- |
| Age, mean±SD, years | 62.70±12.44 | 61.30±16.04 | .830 |
| Male, n (%) | 7(70.00) | 5(50.00) | .650 |
| APACHE II score, mean±SD | 19.20±6.14 | 19.10±8.75 | .977 |
| SOFA score, mean±SD | 6.60±4.16 | 7.10±4.17 | .792 |
| Comorbidity |  |  |  |
| Hypertension, n (%) | 8(80.00) | 5(50.00) | .350 |
| Diabetes, n (%) | 5(50.00) | 3(30.00) | .650 |
| Others, n (%) | 2(20.00) | 4(40.00) | .628 |
| Infection site |  |  | .855 |
| Upper gastrointestinal tract, n (%) | 3(30.00) | 3(30.00) |  |
| Lower gastrointestinal tract, n (%) | 2(20.00) | 3(30.00) |  |
| Others, n (%) | 5(50.00) | 4(40.00) |  |

Abbreviations: SALD, sepsis-associated liver dysfunction; SIC, sepsis-induced cholestasis; APACHE II, Acute Physiology and Chronic Health Evaluation II; SOFA, Sequential Organ Failure Assessment; SD, standard deviation.

**Supplementary Table 3.** The main taxa at the phylum and genus levels between non-SALD and SIC groups at days 1, 3, and 7 of ICU admission

|  | Non-SALD group (n=10) | | |  | SIC group (n=10) | | |
| --- | --- | --- | --- | --- | --- | --- | --- |
|  | d1 | d3 | d7 |  | d1 | d3 | d7 |
| Alpha diversity |  |  |  |  |  |  |  |
| Observed OTUs, median (IQR) | 195.00(152.00, 229.75) | 180.00(148.00, 245.5) | 304.00(165.75, 368.75) |  | 226.00(153.75, 304.75) | 236.00(121.25, 301.50) | 185.00(118.25, 214.75)^c^ |
| Chao1, median (IQR) | 195.47(159.32, 231.94) | 184.50(154.06, 246.60) | 305.29(165.97, 369.10) |  | 234.17(154.31, 305.12) | 236.61(127.52, 305.12) | 188.94(123.11, 217.79)^c^ |
| Shannon, median (IQR) | 3.48(2.19, 5.73) | 4.67(2.03, 5.28) | 5.91(3.54, 6.57) |  | 4.07(2.93, 5.31) | 2.99(1.89, 4.11) | 2.56(1.63, 4.11)^c^ |
| Simpson, median (IQR) | 0.82(0.64, 0.96) | 0.91(0.64, 0.95) | 0.97(0.76, 0.98) |  | 0.86(0.68, 0.92) | 0.63(0.50, 0.87) ^b^ | 0.65(0.39, 0.88)^c^ |
| Pielou_e, median (IQR) | 0.48(0.29, 0.73) | 0.62(0.29, 0.68) | 0.71(0.48, 0.78) |  | 0.57(0.38, 0.64) | 0.38(0.29, 0.50) | 0.34(0.22 ,0.55)^c^ |
| Mean relative abundance of Phylum taxa |  |  |  |  |  |  |  |
| *Firmicutes*, (%) | 36.80 | 44.76 | 59.88 |  | 26.95 | 33.95 | 27.36^c^ |
| *Bacteroidetes*, (%) | 10.39 | 17.73 | 20.43 |  | 14.97 | 6.68 | 8.32^c^ |
| *Proteobacteria*, (%) | 43.46 | 21.23 | 9.66 |  | 47.37 | 56.33^b^ | 57.89^c^ |
| *Chloroflexi*, (%) | 0.01 | 0.00 | 0.00 |  | 0.01 | 0.01^b^ | 0.00 |
| *Cyanobacteria*, (%) | 0.01 | 0.00 | 0.11 |  | 0.06 | 0.16^b^ | 0.03 |
| *Actinobacteria*, (%) | 4.28 | 7.10 | 5.47 |  | 7.62 | 2.10 | 5.77^c^ |
| *Fusobacteria*, (%) | 2.49 | 7.57 | 1.06 |  | 0.41 | 0.03 | 0.05^c^ |
| Mean relative abundance of genus taxa |  |  |  |  |  |  |  |
| *Enterococcus*, (%) | 14.69 | 19.99 | 17.48 |  | 1.29^a^ | 9.87 | 20.84 |
| *Megasphaera*, (%) | 0.02 | 0.32 | 0.39 |  | 0.97 | 0.06 | 1.16 |
| *Collinsella*, (%) | 0.07 | 2.07 | 0.71 |  | 0.28^a^ | 0.03 | 0.01 |
| *Ruminococcus_2*, (%) | 0.00 | 0.28 | 0.12 |  | 0.10^a^ | 0.02 | 0.01 |
| *Ruminococcaceae_unclassified*, (%) | 0.07 | 0.26 | 0.92 |  | 0.01^a^ | 0.24 | 0.10 |
| *Megamonas*, (%) | 2.23 | 5.70 | 1.61 |  | 0.01 | 0.00^b^ | 0.07 |
| *Hungatella*, (%) | 0.50 | 0.98 | 0.25 |  | 0.68 | 0.01^b^ | 0.01^c^ |
| *Prevotella_2*, (%) | 0.02 | 0.90 | 0.61 |  | 0.00 | 0.00^b^ | 0.00 |
| *Ruminococcaceae_UCG-002*, (%) | 0.79 | 0.77 | 1.13 |  | 0.451 | 0.07^b^ | 0.01^c^ |
| *Sphingopyxis*, (%) | 0.41 | 0.13 | 0.05 |  | 0.31 | 0.56^b^ | 0.46^c^ |
| *Burkholderia-Caballeronia-Paraburkholderia*, (%) | 8.22 | 5.44 | 1.40 |  | 9.45 | 17.17 | 31.13^c^ |
| *Delftia*, (%) | 1.09 | 2.34 | 0.04 |  | 3.01 | 1.81 | 4.60^c^ |
| *Lactobacillus*, (%) | 1.91 | 0.12 | 1.66 |  | 6.72 | 6.99 | 0.15^c^ |
| *Lachnoclostridium*, (%) | 1.29 | 1.88 | 1.11 |  | 0.63 | 0.32 | 0.24^c^ |
| *Anaerococcus*, (%) | 0.07 | 0.41 | 0.80 |  | 1.05 | 0.05 | 0.01^c^ |

Abbreviations: SALD, sepsis-associated liver dysfunction; SIC, sepsis-induced cholestasis; OTUs, operational taxonomic units; IQR, interquartile range; ^a^, compared to corresponding indicators on day 1 in non-SALD group; ^b^, compared to corresponding indicators on day 3 in non-SALD group; ^c^, compared to corresponding indicators on day 7 in non-SALD group.

## Supplementary Figures

**A**

**
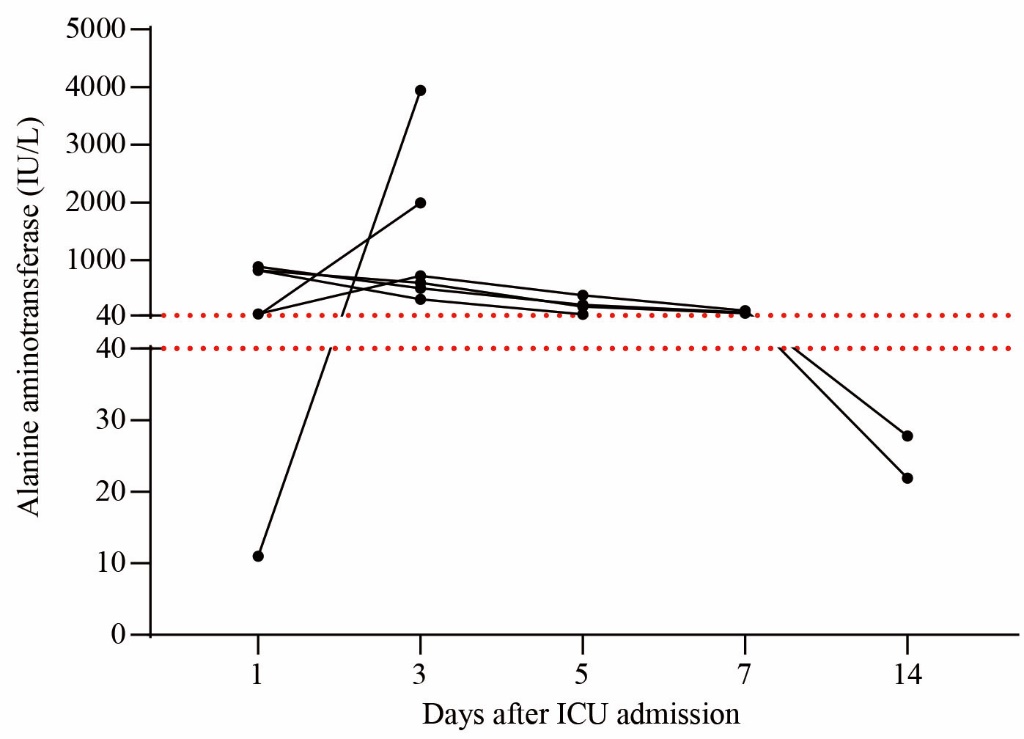
**

**B**

**
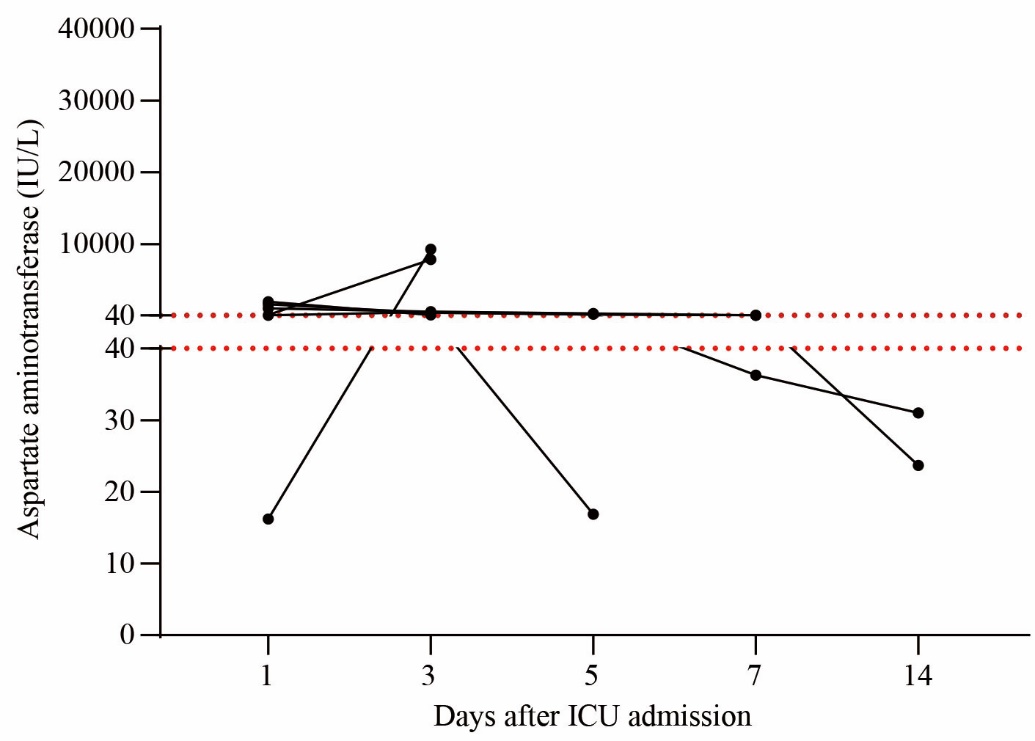
**

**C**

**
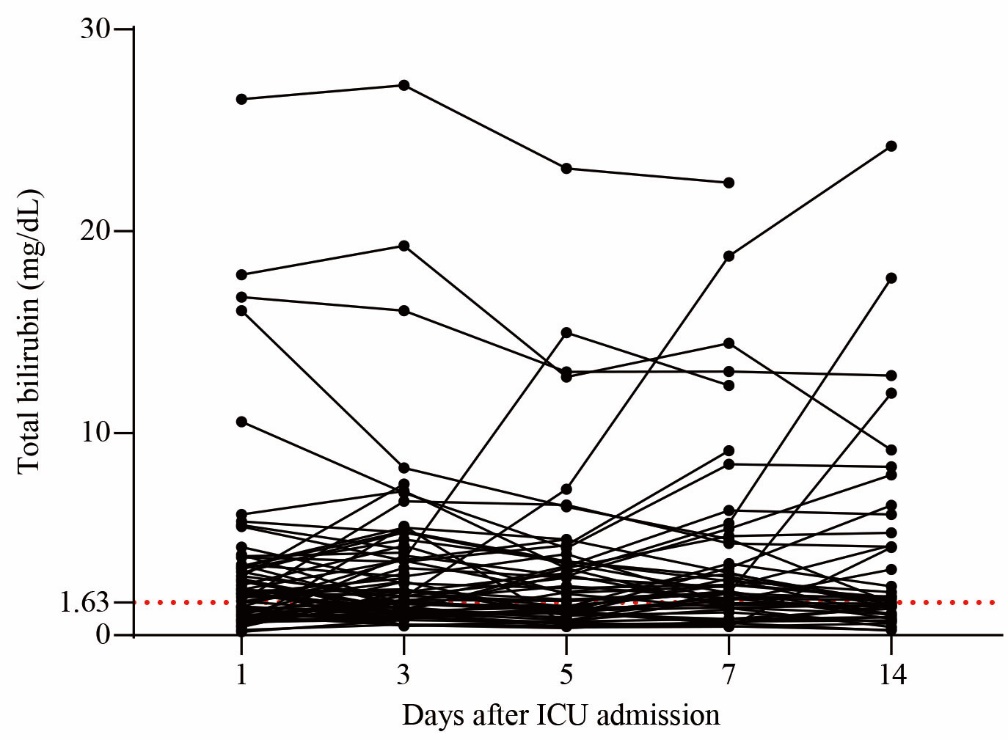
**

**Supplementary Figure 1.** Trend of serum transaminase and TBIL levels in patients with SALD. **(A)** The trend of serum ALT in patients with hypoxic hepatitis. **(B)** The trend of serum AST in patients with hypoxic hepatitis. (**C**) The trend of serum TBIL in patients with SIC. TBIL, total bilirubin; SALD, sepsis-associated liver dysfunction; ALT, alanine aminotransferase; AST, aspartate aminotransferase; SIC, sepsis-induced cholestasis; red dotted lines depict the upper limit of normal values.

**
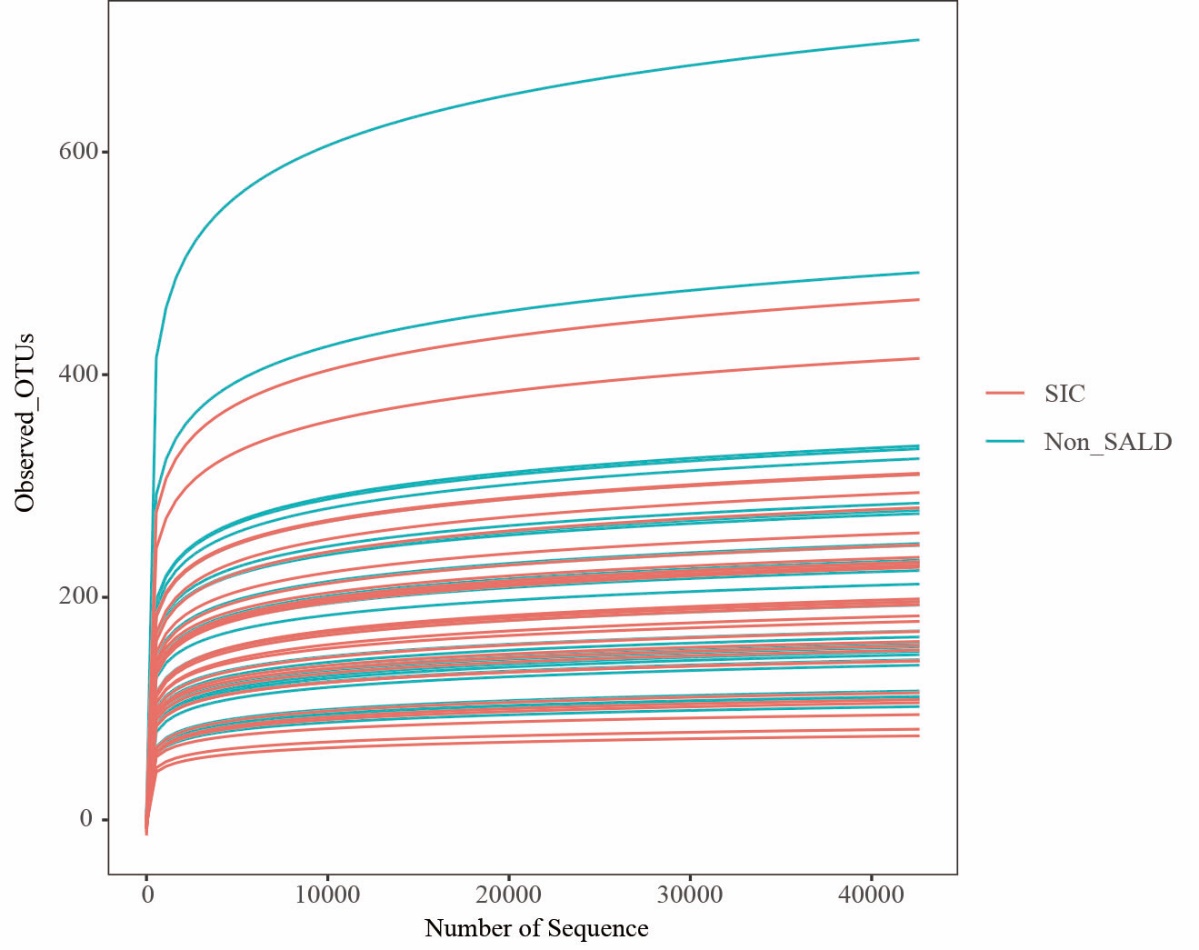
**

**Supplementary Figure 2.** The rarefaction curves of 16S sequencing data in the entire population. SIC, sepsis-induced cholestasis; OTUs, operational taxonomic units.

**
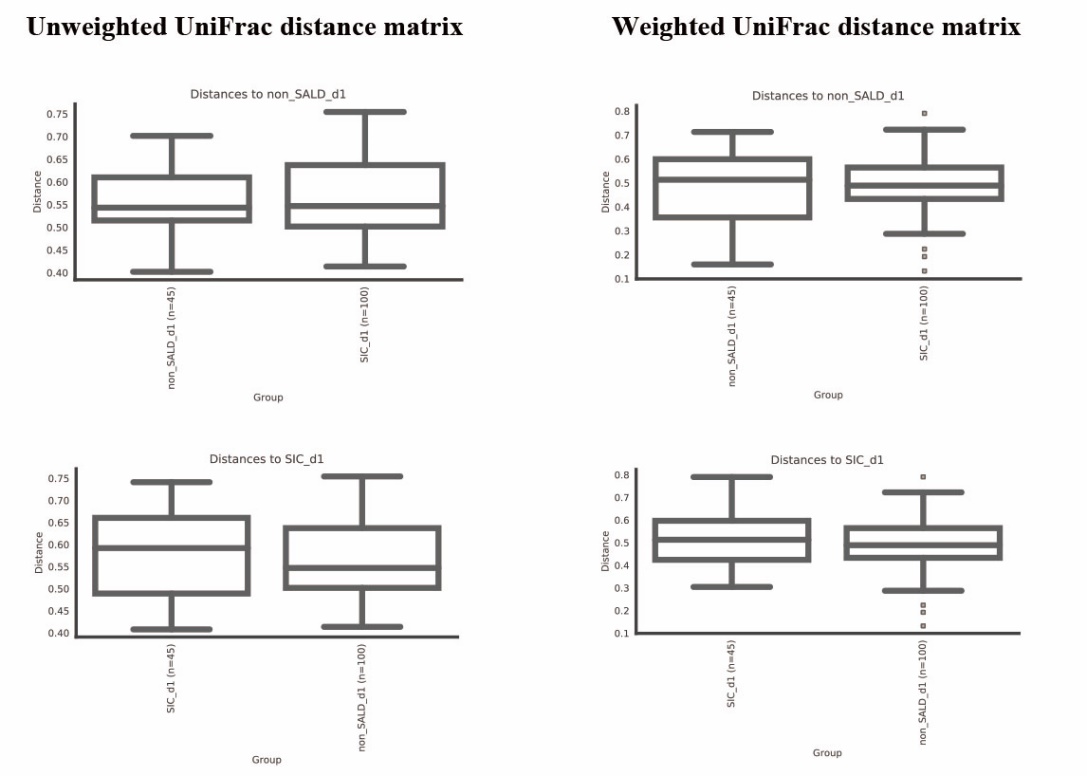
**

**Supplementary Figure 3.** ANOSIM analysis in unweighted and weighted UniFrac distance metrics between non-SALD and SIC groups on day 1 after ICU admission**.** ANOSIM, analysis of similarities; SALD, sepsis-associated liver dysfunction; SIC, sepsis-induced cholestasis; ICU, intensive care unit; non_SALD_d1 represents day 1 in non-SALD group; SIC_d1 represents day 1 in SIC group.

**
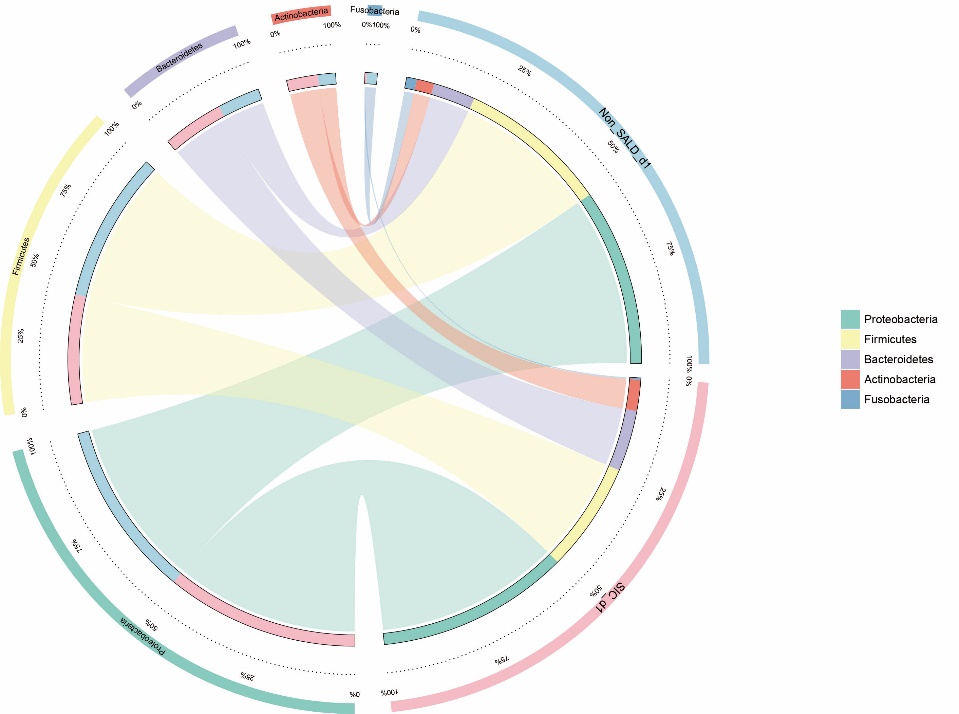
**

**Supplementary Figure 4.** The Circos plot of top five phylum taxonomic levels on day 1 after ICU admission in the non-SALD and SIC groups**.** ICU, intensive care unit; SALD, sepsis-associated liver dysfunction; SIC, sepsis-induced cholestasis; non_SALD_d1 represents day 1 in non-SALD group; SIC_d1 represents day 1 in SIC group.


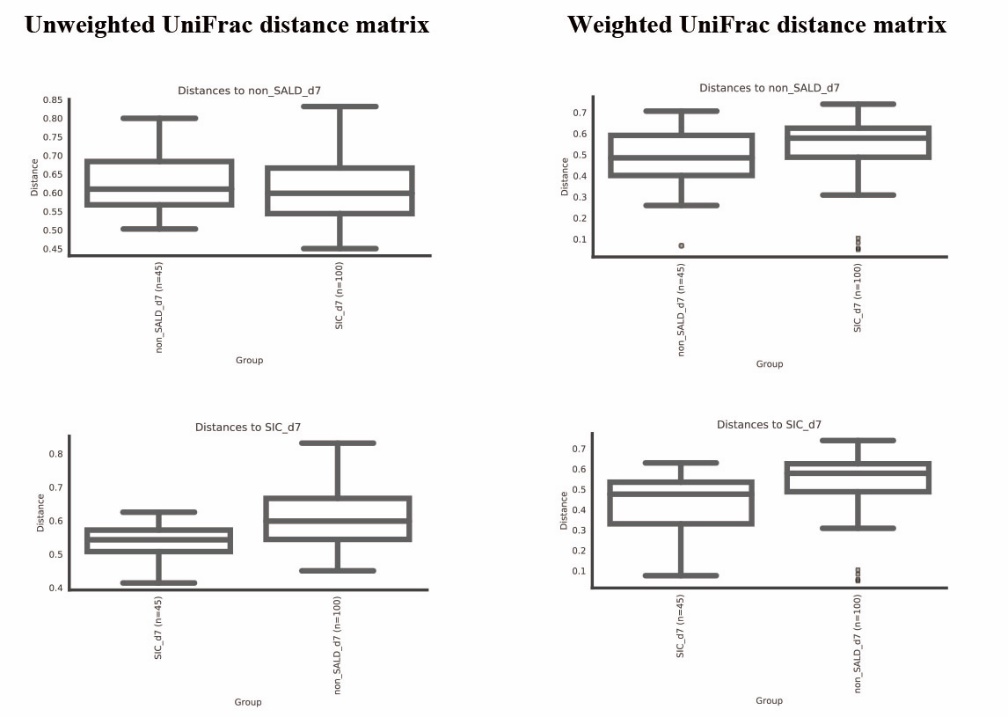


**Supplementary Figure 5.** ANOSIM analysis in unweighted and weighted UniFrac distance metrics between non-SALD and SIC groups on day 7 after ICU admission. ANOSIM, analysis of similarities; SALD, sepsis-associated liver dysfunction; SIC, sepsis-induced cholestasis; ICU, intensive care unit; non_SALD_d7 represents day 7 in non-SALD group; SIC_d7 represents day 7 in SIC group.

**
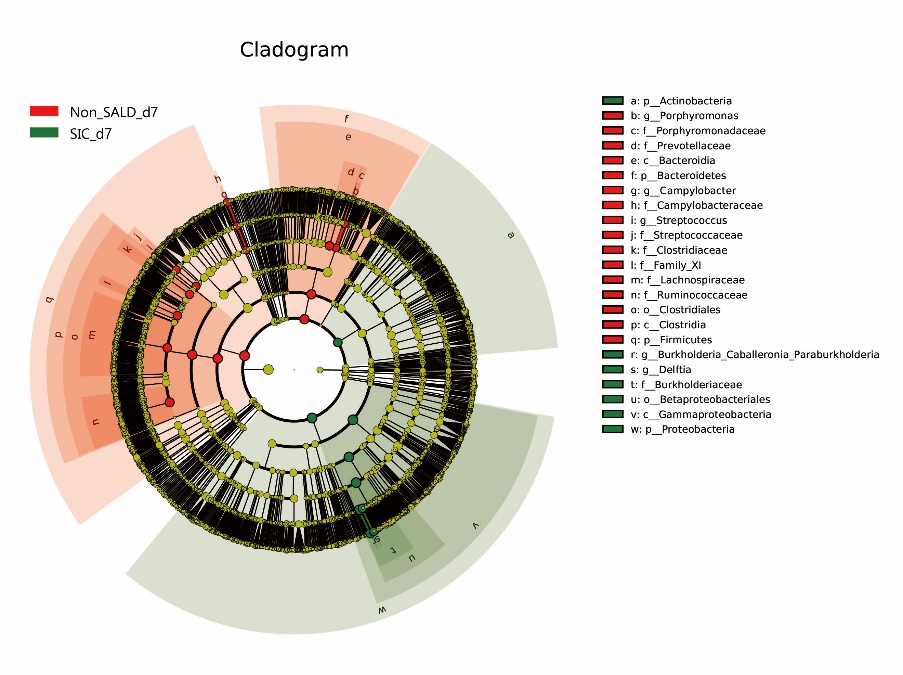
**

**Supplementary Figure 6.** A cladogram depicting differentially abundant taxa analyzed using LEfSe between non-SALD and SIC groups on day 7 after ICU admission. LEfSe, linear discriminant analysis effect size; SALD, sepsis-associated liver dysfunction; SIC, sepsis-induced cholestasis; ICU, intensive care unit; non_SALD_d7 represents day 7 in non-SALD group; SIC_d7 represents day 7 in SIC group.


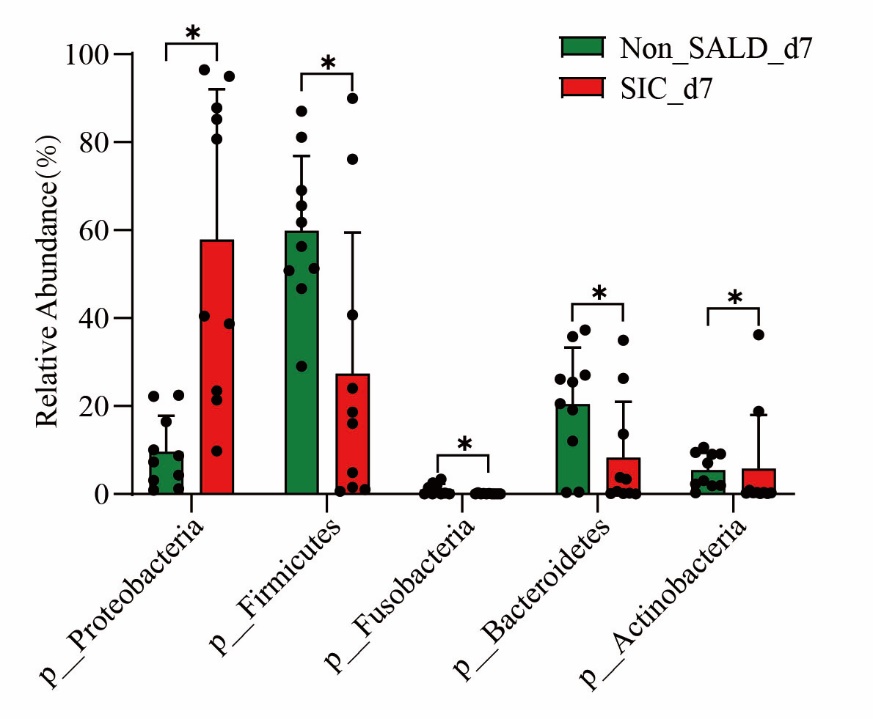


**Supplementary Figure 7.** Comparison of the gut microbiota composition at the phylum level among non-SALD and SIC groups on day 7 after ICU admission**.** SALD, sepsis-associated liver dysfunction; SIC, sepsis-induced cholestasis; ICU, intensive care unit; non_SALD_d7 represents day 7 in non-SALD group; SIC_d7 represents day 7 in SIC group; **p*<0.05.


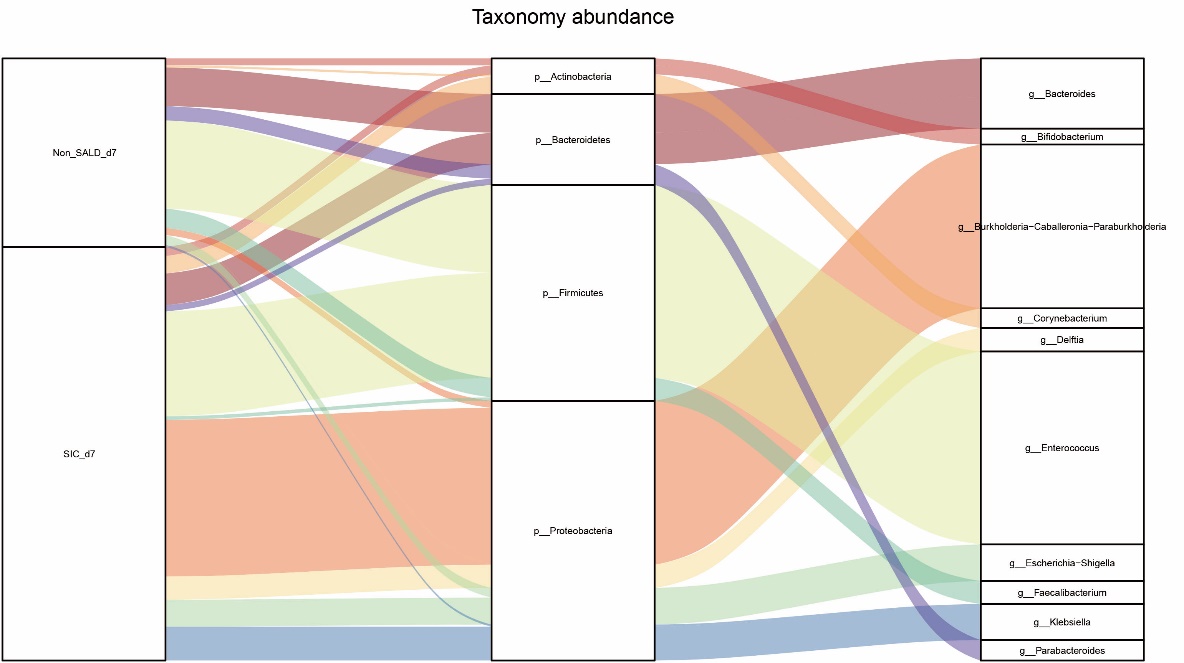


**Supplementary Figure 8.** Sankey plots describing the pairing interactions between the phylum and genus levels in the non-SALD and SIC groups on day 7 after ICU admission. SALD, sepsis-associated liver dysfunction; SIC, sepsis-induced cholestasis; ICU, intensive care unit; non_SALD_d7 represents day 7 in non-SALD group; SIC_d7 represents day 7 in SIC group.

**Non-SALD group:**

**
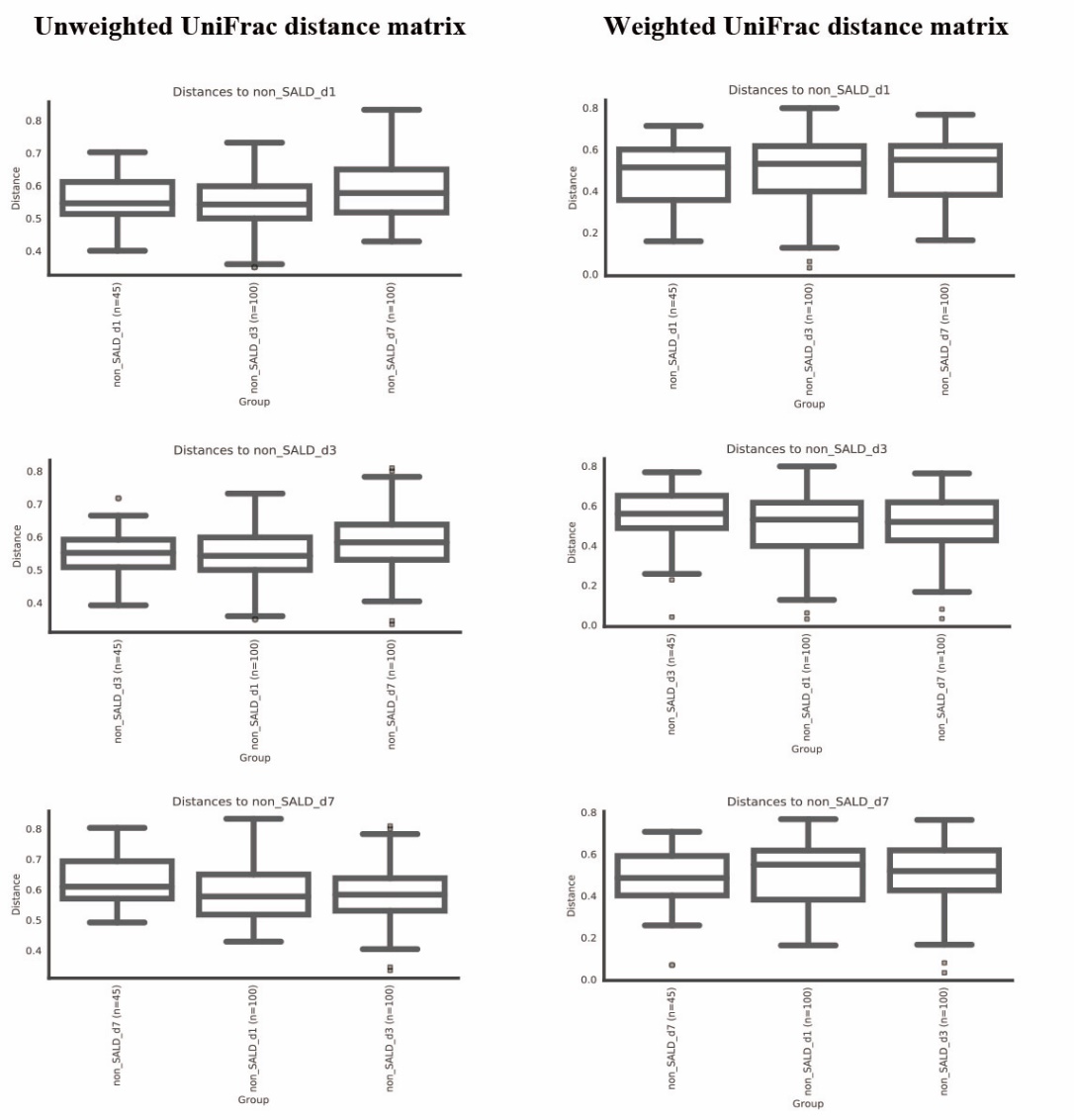
**

**SIC group:**


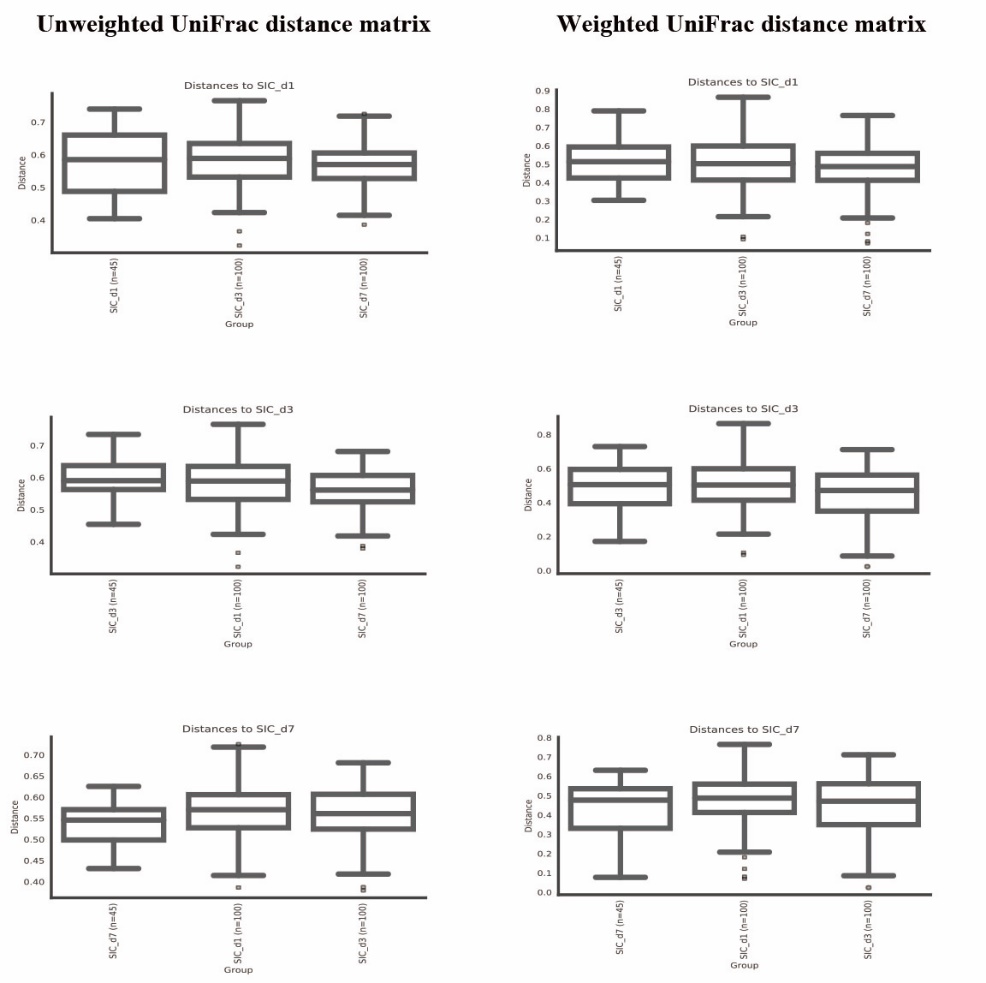


**Supplementary Figure 9.** ANOSIM analysis in unweighted and weighted UniFrac distance metrics among different time points in the non-SALD and SIC groups. ANOSIM, Analysis of similarities; SALD, sepsis-associated liver dysfunction; SIC, sepsis-induced cholestasis; non_SALD_d1 represents day 1 in non-SALD group; non_SALD_d3 represents day 3 in non-SALD group; non_SALD_d7 represents day 7 in non-SALD group; SIC_d1 represents day 1 in SIC group; SIC_d3 represents day 3 in SIC group; SIC_d7 represents day 7 in SIC group.

**Non-SALD group:**

**
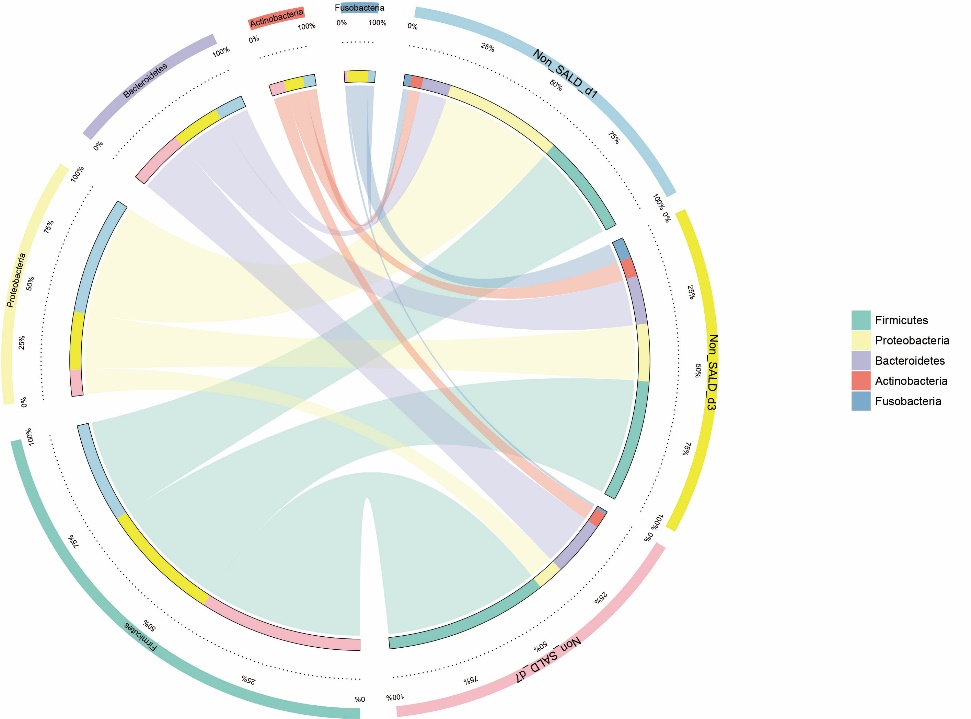
**

**SIC group:**

**
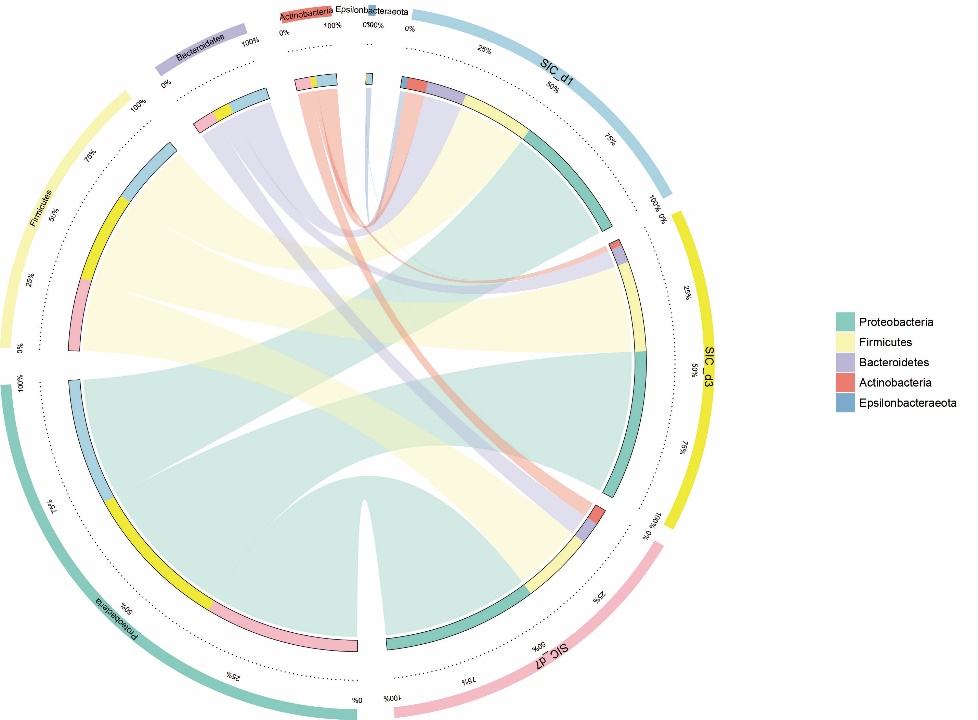
**

**Supplementary Figure 10.** The Circos plot of changes in top five phylum taxonomic levels on day 1, 3, and 7 after ICU admission in the non-SALD and SIC groups. ICU, intensive care unit; SALD, sepsis-associated liver dysfunction; SIC, sepsis-induced cholestasis; non_SALD_d1 represents day 1 in non-SALD group; non_SALD_d3 represents day 3 in non-SALD group; non_SALD_d7 represents day 7 in non-SALD group; SIC_d1 represents day 1 in SIC group; SIC_d3 represents day 3 in SIC group; SIC_d7 represents day 7 in SIC group.
